# Extensive Conserved Synteny of Genes between the Karyotypes of *Manduca sexta* and *Bombyx mori* Revealed by BAC-FISH Mapping

**DOI:** 10.1371/journal.pone.0007465

**Published:** 2009-10-15

**Authors:** Yuji Yasukochi, Makiko Tanaka-Okuyama, Fukashi Shibata, Atsuo Yoshido, František Marec, Chengcang Wu, Hongbin Zhang, Marian R. Goldsmith, Ken Sahara

**Affiliations:** 1 Insect Genome Research Unit, National Institute of Agrobiological Sciences, Tsukuba, Ibaraki, Japan; 2 Laboratory of Applied Molecular Entomology, Graduate School of Agriculture, Hokkaido University, Sapporo, Japan; 3 Institute of Entomology, Biology Centre ASCR, České Budějovice, Czech Republic; 4 Department of Soil and Crop Sciences, Texas A&M University, College Station, Texas, United States of America; 5 Biological Sciences Department, University of Rhode Island, Kingston, Rhode Island, United States of America; Texas A&M University, United States of America

## Abstract

**Background:**

Genome sequencing projects have been completed for several species representing four highly diverged holometabolous insect orders, Diptera, Hymenoptera, Coleoptera, and Lepidoptera. The striking evolutionary diversity of insects argues a need for efficient methods to apply genome information from such models to genetically uncharacterized species. Constructing conserved synteny maps plays a crucial role in this task. Here, we demonstrate the use of fluorescence *in situ* hybridization with bacterial artificial chromosome probes as a powerful tool for physical mapping of genes and comparative genome analysis in Lepidoptera, which have numerous and morphologically uniform holokinetic chromosomes.

**Methodology/Principal Findings:**

We isolated 214 clones containing 159 orthologs of well conserved single-copy genes of a sequenced lepidopteran model, the silkworm, *Bombyx mori*, from a BAC library of a sphingid with an unexplored genome, the tobacco hornworm, *Manduca sexta*. We then constructed a BAC-FISH karyotype identifying all 28 chromosomes of *M. sexta* by mapping 124 loci using the corresponding BAC clones. BAC probes from three *M. sexta* chromosomes also generated clear signals on the corresponding chromosomes of the convolvulus hawk moth, *Agrius convolvuli*, which belongs to the same subfamily, Sphinginae, as *M. sexta*.

**Conclusions/Significance:**

Comparison of the *M. sexta* BAC physical map with the linkage map and genome sequence of *B. mori* pointed to extensive conserved synteny including conserved gene order in most chromosomes. Only a few rearrangements, including three inversions, three translocations, and two fission/fusion events were estimated to have occurred after the divergence of Bombycidae and Sphingidae. These results add to accumulating evidence for the stability of lepidopteran genomes. Generating signals on *A. convolvuli* chromosomes using heterologous *M. sexta* probes demonstrated that BAC-FISH with orthologous sequences can be used for karyotyping a wide range of related and genetically uncharacterized species, significantly extending the ability to develop synteny maps for comparative and functional genomics.

## Introduction

Great diversity, one of the most striking characteristics of insects, presents a serious challenge for genomics, and thus constructing a firm foundation for comparative genomics of insects is of critical importance [1]. Genome sequencing projects have been completed for several species representing four highly diverged holometabolous insect orders, Diptera, Hymenoptera, Coleoptera, and Lepidoptera [2]–[7]. This is, however, far from sufficient in view of the important role of insects in diverse ecosystems, great number of insect species affecting human activities as pests or vectors of diseases, and need for sequence data for studies on processes such as insect evolution, ecology, behavior, insecticide resistance, development, and physiology. In spite of recent progress in the development of faster, more cost-effective sequencing technologies than used in the first genome projects [8], [9], it is still unrealistic to sequence all species of interest *de novo*. This particularly applies to Lepidoptera (moths and butterflies), one of the most diverse and species-rich group of insects, comprising the second largest order of animals with more than 150,000 named species. A partial solution is to study widely conserved molecular mechanisms underlying basic characters and processes in model species as a guide for investigating related phenomena in less well-studied relatives. Determining orthologous genes in multiple model species will greatly enhance knowledge of evolutionary patterns and processes [10], and promote research in non-sequenced species.

Many inherited phenotypes and behaviors are restricted to a narrow range of species, and are not available or difficult to study in the sequenced models. Ultimately, positional cloning may be the only way to reveal distinctive underlying molecular mechanisms. Although a difficult task in non-sequenced species where a chromosome region responsible for the phenotype of interest is poorly defined, it can be greatly facilitated by establishing comparative maps. Providing that the location and order of genes are conserved, positional information from orthologous chromosomal regions of well-characterized model species will save the time and expense required for the analysis of species with unexplored genomes. Loci of interest can then be identified and recovered by reference to common flanking markers [11].

Significant preliminary evidence indicates that genome organization at the chromosome level is well conserved between the silkworm, *Bombyx mori*, representing the Bombycoidea, and two nymphalid butterflies representing the Papilionoidea, *Heliconius melpomene* [12], [13] and *Bicyclus anynana* [14]. Although local gene rearrangements have occurred among these groups of Lepidoptera, using the *B. mori* genome as a reference was shown in these reports to be an effective approach for initial investigation of the evolution of gene organization and arrangement, at least for Macrolepidoptera.

The tobacco hornworm, *Manduca sexta* (Sphingidae), also belongs to the superfamily Bombycoidea. For years this species has been used as a favorite experimental animal in insect biochemistry, physiology, and especially in neurobiology [15]–[17]. Sequence and expression data for *M. sexta* genes has been increasing [18]–[20]. Above all, 8,344 expressed sequence tags (ESTs) have been deposited in Genbank [21], [22], and bacterial artificial chromosome (BAC) libraries have been constructed [23]. Yet, despite its widespread use in research, little is known about its genome. Until recently, the haploid chromosome number (n = 28) was known only from a conference report [24], but no detailed chromosome analysis has been performed and no linkage maps have been constructed. Such an imbalance between molecular data and genetic knowledge seriously impedes progress in characterizing links between phenotype and genotype in an important model organism. To fill this gap, we initiated research on the synteny between chromosomes of *B. mori* and *M. sexta* by comparatively mapping orthologous genes with the help of BAC-FISH (fluorescent *in situ* hybridization with BAC probes).

Lepidoptera have small, numerous, and morphologically uniform holokinetic chromosomes, which are refractory to differential cytogenetic techniques. In our previous work [25], [26], we showed that the application of BAC-FISH technology can provide specific recognition of individual lepidopteran chromosomes and is also a powerful tool for physical mapping of genes on pachytene chromosomes, which allow much higher resolution than tiny and compact metaphase chromosomes. In our first study, we confirmed the chromosome number in *M. sexta*, introduced five-color BAC-FISH to facilitate mapping, and used the improved BAC-FISH protocol to show broadly conserved synteny of genes including their order between *B. mori* chromosome 15 and the corresponding chromosome of *M. sexta* [26].

In the present study, we isolated additional *M. sexta* BAC clones containing 159 conserved genes by means of PCR-based screening. We then mapped the BAC clones by FISH to pachytene chromosomes, constructed a complete BAC-FISH karyotype of *M. sexta*, and by comparison with *B. mori* linkage groups, examined conserved synteny of genes between the two species. We also showed the applicability of BAC-FISH to a related species by cross-hybridization of *M. sexta* BAC probes to selected chromosomes of the convolvulus hawk moth, *Agrius convolvuli*, a representative of the same subfamily, Sphinginae, but from a different tribe (Acherontiini) than *M. sexta* (Sphingini) [27]. The fact that heterologous BAC probes can be used for karyotyping and gene mapping in related species without any previous genomic knowledge represents additional significant benefits of BAC-FISH.

## Results

### Mapping of conserved genes in *Bombyx mori*

We previously showed that the gene order is well conserved between chromosome 15 of *B. mori* and the corresponding chromosome of *M. sexta* by mapping *M. sexta* orthologs of six genes using BAC-FISH [26]. Using the same strategy, we designed experiments to identify relationships between additional chromosomes of *B. mori* and *M. sexta*.

DNA sequences of genes and ESTs of *M. sexta* were used to find single-copy orthologs in *B. mori* by TBLASTN search against WGS genome sequences [28], [29]. More than 600 candidates were identified, some of which had been directly mapped onto a linkage map of *B. mori* or localized to the mapped BAC contigs in our previous report [12]. We subsequently designed 86 pairs of new polymorphic STS primers for unmapped *B. mori* orthologs (Table S1) and localized them using 166 F_2_ progeny. In all, we identified chromosomal locations of 292 putative single-copy conserved genes in *B. mori* (Table S2).

### Isolation of *Manduca sexta* BAC clones containing conserved genes

To isolate BAC clones of *M. sexta* using PCR-based screening, we designed primer sets from *M. sexta* genes for mapped *B. mori* orthologs. Ultimately, 159 STS primers encompassing from three to nine loci for each *B. mori* chromosome (Table S3) could be used for PCR-based screening (Table S4).

For rapid characterization of the overall synteny, minimal initial screening of the BAC library was carried out, and, in principle, we did not isolate multiple clones for each STS. Instead, a second clone was isolated only when an initially isolated clone was found to be unsuitable as a FISH probe, for such reasons as generating weak or non-specific signals. In all, 214 BACs were isolated corresponding to the 159 STSs. Of these, nine pairs and two triplets of STSs for which orthologs were closely spaced in *B. mori* were positive for identical clones (Table S3), thus indicating microsynteny, i.e., conserved fine scale gene order, between the two species.

### BAC-FISH karyotype of *Manduca sexta*

**Figure 1:**
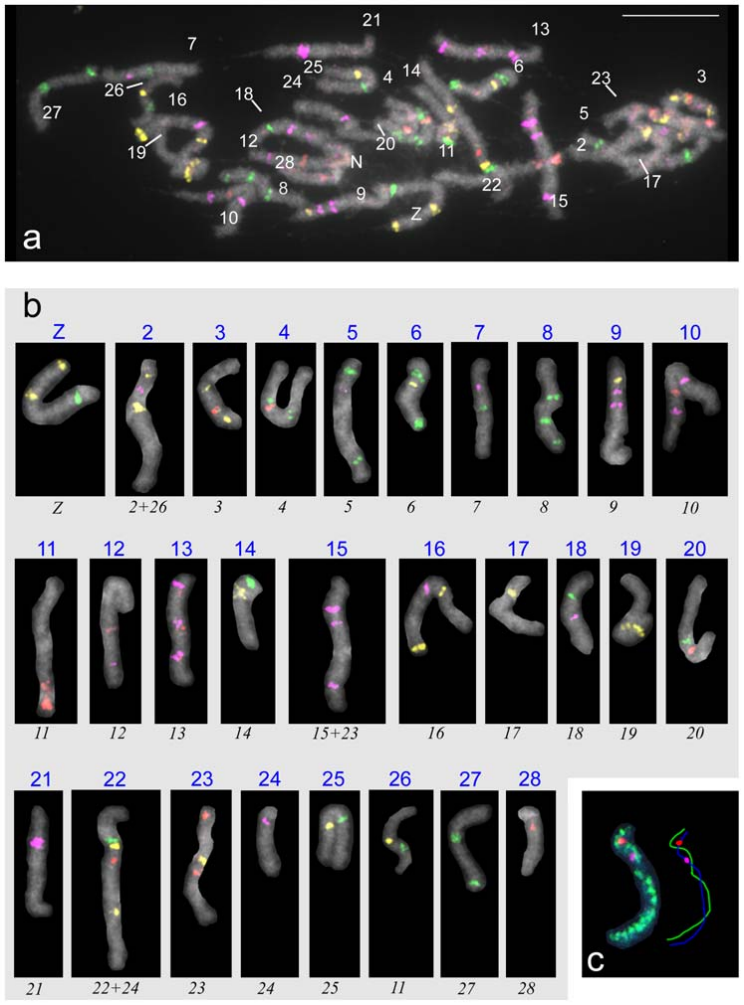
BAC-FISH pachytene karyotype of *Manduca sexta*. (a) A spermatocyte pachytene complement of n = 28 bivalents, each showing 1–3 pseudocolored hybridization signals of individual BAC probes. (b) Pachytene bivalents of the same complement as in (a) arranged according to corresponding *B. mori* chromosome numbers (black italic numbers). (c) Identification of the sex chromosome bivalent WZ in a pachytene oocyte by genomic *in situ* hybridization (GISH) combined with BAC-FISH. With GISH, the female genomic probe (green signals) highlighted the whole W-chromosome thread of the WZ bivalent, while hybridization signals of the 42G01 (red) and 22D05 (cyan) BAC clones marked the Z-chromosome thread. Chromosomes were stained with DAPI fluorochrome; BAC probes were labeled with Green-dUTP (green), Orange-dUTP (yellow), Red-dUTP (red), and Cy5-dUTP (cyan). LG, linkage group of *B. mori*; N, nucleolus; Z, sex chromosome bivalent (ZZ). For details, see Table S5. Bar  = 10 µm.

To elucidate the gross relationship of chromosomes between *B. mori* and *M. sexta*, we sought to identify all 28 chromosomes of *M. sexta* by BAC-FISH using several combinations of BAC probes. We selected BAC probes based primarily on the chromosomal location of orthologous genes in *B. mori* (Table S5). After BAC-FISH sorting on each of 28 bivalents, we hybridized a cocktail of 63 BAC probes to male chromosome preparations of *M. sexta*. On the first attempt, we clearly identified 20 bivalents in a pachytene complement (Table S5), but could not completely distinguish the bivalents of chromosomes Z and 19, 12 and 13, 14 and 17, and 18 and 24 from each other. Therefore, we carried out another round of BAC-FISH with additional probes using a recently developed reprobing protocol designed for Lepidoptera [30]. Since we had already identified the location of BACs 18A03, 28D18, 05H01, and 47E07 on chromosomes Z, 13, 14, and 18, respectively, we used them for the reprobed FISH. With the help of the second round of BAC-FISH we successfully defined all 28 individual bivalents (Fig. 1a). The identification of all chromosomes at once in a single pachytene complement enabled us to assemble a complete male *M. sexta* karyotype (Fig. 1b). We confirmed the correct classification of the Z chromosome as the sex chromosome on spread pachytene oocytes by genomic *in situ* hybridization (GISH) performed in combination with two BAC probes derived from the putative Z chromosome (Fig. 1c). This enabled us to visualize the WZ sex chromosome bivalent by strong binding of the female genomic probe to the W chromosome, while hybridization signals on other chromosomes were weakened by the excess of unlabeled male genomic DNA used as a competitor [31].

*M. sexta* is thus the second species after *B. mori* [25] with a completely described karyotype in Lepidoptera. At first sight, the structure of the *M. sexta* karyotype indicates extensive conserved synteny of genes between chromosomes of *M. sexta* and *B. mori*, with only a few rearrangements detected as described below.

### Conserved synteny and order of orthologous genes between *Manduca sexta* and *Bombyx mori*

**Figure 2:**
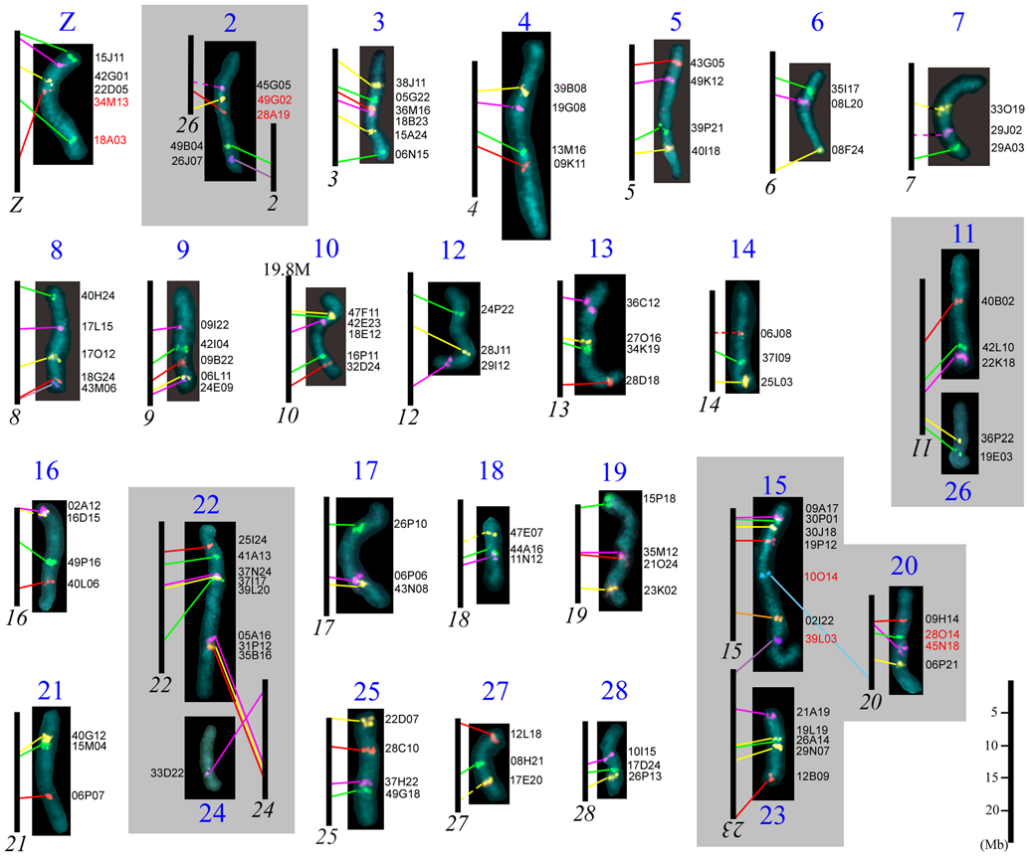
Genome wide comparison of orthologous genes between individual chromosomes of *Manduca sexta* and *Bombyx mori*. DAPI-stained images of individual pachytene bivalents of *M. sexta* (blue numbers) show hybridization signals of BAC probes. Vertical bars represent corresponding parts of *B. mori* chromosomes (black italic numbers) drawn to relative scale in Mb taken from Kaikobase. Individual images of chromosome bivalents were obtained from different pachytene complements and/or from different preparations; therefore, their lengths differed depending on the stage and do not reflect relative sizes of the bivalents (for relative sizes, see Fig. S1). BAC probe codes are shown on the right of each chromosome image (for details, see Table S5).

We carried out a detailed BAC-FISH analysis for each pachytene bivalent of *M. sexta* using BAC probes carrying orthologs of *B. mori* genes in order to identify the extent of synteny, including conserved gene order, and to detect chromosome rearrangements. Our experiments confirmed a well-conserved synteny of genes between *M. sexta* and *B. mori* in 20 chromosomes (Fig. 2). Among these, the gene order was identical in 19 chromosomes.

The only exception was the Z chromosome, in which BAC-FISH signals demonstrated conserved order of three orthologous genes (all located at about one third of chromosome length) but inverted order of two other orthologs. This indicated the presence of a large inversion comprising about half of the Z chromosome. Nevertheless, due to conserved synteny we designated each of the 20 chromosomes of *M. sexta* according to the corresponding chromosome of *B. mori* (Fig. 2, Table S5).

In the remaining 8 chromosomes of *M. sexta*, BAC-FISH mapping revealed interchromosomal rearrangements relative to the karyotype of *B. mori*. We found that one chromosome of *M. sexta*, designated as chromosome 2, was composed of chromosome 2 and chromosome 26 of *B. mori*; in addition, the latter part exhibited an inverted order of two orthologous genes. On the other hand, chromosome 11 of *B. mori* was composed of chromosomes 11 and 26 of *M. sexta* (Fig. 2, Table S5). These differences can be explained simply by two events involving three chromosomes that occurred after the split of Sphingidae and Bombycidae from a common ancestor: namely, by fusion of two chromosomes into one chromosome plus fission of another chromosome into two chromosomes. As our data suggest these rearrangements preserved the ancestral order of genes except for one smaller segment, in which an inversion occurred (cf. chromosome 26 of *B. mori* and chromosome 2 of *M. sexta* in Fig. 2). Rearrangements between chromosomes 22 and 24, and between chromosomes 15 and 23 obviously resulted from a simple translocation of terminal chromosome segments (Fig. 2). In addition, we identified a translocation between chromosomes 15 and 20, which was not apparent from our initial *M. sexta* karyotyping (cf. Figs. 1 and 2). Thus, *M. sexta* chromosome 15 contained, besides the whole chromosome 15 of *B. mori*, orthologs of *B. mori* genes located at one end of chromosome 23 and an ortholog of the ribosomal protein S20 gene located at one end of *B. mori* chromosome 20. Finally, BAC-FISH revealed an inversion that altered gene order in chromosome 20 of *M. sexta* and *B. mori* (Fig. 2).

Taken together, we confirmed an extensive conserved synteny of genes between chromosomes of *M. sexta* and *B. mori*, including a highly conserved gene order. A total of 124 out of 131 orthologous genes mapped in this study retained the same order in both species. BAC-FISH mapping revealed only eight chromosomal rearrangement events in the karyotype evolution of the two species that disturbed the synteny (five events) or the order of orthologous genes (three events) (Fig. 2, Table S5). Relative positions of the BAC-FISH signals on *M. sexta* chromosomes are shown in Fig. S1.

### Application of *Manduca sexta* BAC probes to *Agrius convolvuli*

**Figure 3:**
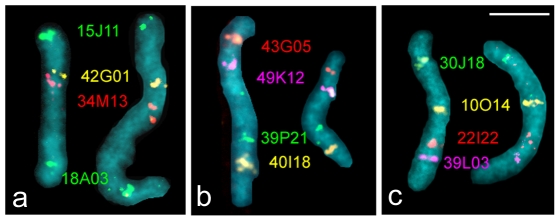
Cross-hybridization of *Manduca sexta* BAC probes to male pachytene bivalents of *Agrius convolvuli*. (a) Z-chromosome bivalents. (b) Chromosome 5 bivalents. (c) Chromosome 15 bivalents. Each image shows an *M. sexta* bivalent (left) and an *A. convolvuli* bivalent (right) using the same set of *M. sexta* BAC probes. Note that in each image the *A. convolvuli* bivalent shows hybridization signals in the same sequence as the corresponding *M. sexta* bivalent, thus indicating well conserved synteny including the gene order between the two species. BAC probe codes are shown between the bivalents (for details, see Table S5). Bar  = 5 µm.

To test the effectiveness of our cytogenetic mapping technique on a related species, we carried out BAC-FISH on the convolvulus hawk moth, *A. convolvuli* (Sphinginae, Acherontiini). We used *M. sexta* BAC probes that mapped to three representative chromosomes, including the sex chromosome Z and two autosomes (chromosomes 5 and 15) selected as examples without and with rearrangement compared to *B. mori*. All twelve BAC probes derived from *M. sexta* cross-hybridized on the corresponding *A. convolvuli* chromosomes (Fig. 3), indicating that the underlying sequences are well-enough conserved to allow the generation of specific signals, despite the species differences. Additionally, the order of the hybridization signals was identical to the corresponding *M. sexta* genes, which strongly suggests extensive collinearity between *M. sexta* and *A. convolvuli*.

We also tested whether the same *M. sexta* BAC probes could cross-hybridize on *B. mori* chromosomes and the orthologous *B. mori* BAC probes could cross-hybridize on *M. sexta* chromosomes. However, no specific signals were detected in either combination, indicating that the sequence similarity between *B. mori* and *M. sexta* is insufficient for cross-hybridization (Fig. S2).

## Discussion

### Advantages of BAC-FISH karyotyping compared with linkage analysis

Theoretically, mapping of genes in sexually reproducing organisms can be accomplished by linkage analysis. A comparison of linkage maps constructed in different species is feasible and reliable, provided that a sufficient number of orthologous genes are mapped. In Lepidoptera, with the majority of species possessing around thirty pairs of chromosomes [32], more than 100 loci are needed to yield 3–4 loci per linkage group. However, highly polymorphic molecular markers used for most molecular linkage maps are not suitable for comparison of different species. Moreover, it is generally difficult to map conserved genes by classical genetic methods, because “conserved” inevitably means lower rates of polymorphism required for linkage analysis.

Among the molecular linkage maps constructed in Lepidoptera, most employ anonymous markers such as AFLPs and microsatellites [33]–[36]; consequently, only *H. melpomene* (n = 22) and *B. anynana* (n = 28), both Nymphalidae, have been examined for genome-wide synteny to *B. mori*. Seventy-two loci orthologous to *B. mori* genes were used to examine synteny in *H. melpomene* after constructing foundation maps composed of AFLPs and microsatellites [37] and then populating them with gene loci based on cleaved amplified polymorphic sequences derived primarily from ESTs [13]. Similarly, the location of 462 *B. mori* orthologs were compared in *B. anynana*, which has the most gene-rich published lepidopteran linkage maps apart from *B. mori*. However, localizing the orthologs first required construction of a map of 768 SNPs derived from more than 100,000 ESTs [14].

In comparison with such labor-intensive linkage and recombination analysis, the BAC-FISH mapping performed in the present study seems to be more efficient. In a species with a little-known genome, *M. sexta*, we mapped 124 loci by using BAC clones as probes. This enabled us to construct a BAC-FISH karyotype and sufficiently cover all 28 chromosomes with markers for comparative studies. Besides highly reliable and efficient localization of genes, the use of BAC-FISH has several other advantages. First, it does not require polymorphism in the genes examined, whereas it is essential for linkage mapping. Second, BAC-FISH mapping is independent of genetic recombination, facilitating analysis of genomes, chromosomes or chromosomal regions with rare crossovers. Third, it does not require the use of numerous sibs from matings between genetically homogeneous strains to determine the gene order unambiguously. Selection of only 67 *M. sexta* BACs carrying mapped *B. mori* orthologs was sufficient for the identification of all 28 bivalents in a pachytene complement (Fig. 1a) and to allow subsequent karyotyping (Fig. 1b). Finally, mating of the target organism is not required for BAC-FISH-based mapping, whereas it is needed to generate a mapping population for linkage analysis. Independence of recombination, polymorphism and mapping population also mean that a relatively small number of heterogeneous insects collected from wild populations can be analyzed directly using BAC probes established from a standard strain.

Our results with *A. convolvuli* clearly show that *M. sexta* BAC probes can be used for comparative mapping in non-congeneric species. The genus *Agrius* belongs to the tribe Acherontiini, a monophyletic clade within the same subfamily, Sphinginae, as *M. sexta*, which has been classified into the tribe Sphingini. However, a recent molecular phylogeny of sphingids revealed that the clade Sphingini is paraphyletic and that the clade Acherontiini is a sister group of ‘Old World Sphingini,’ whereas the majority of species including *M. sexta* belong to a separate clade consisting mainly of ‘New World’ hawkmoths [27]. This suggests a more distant relationship between *A. convolvuli* and *M. sexta* than previously thought and points out the potential of heterologous BAC-FISH for a wide range of related species. Similar cross-species hybridization of BAC probes was previously demonstrated in mammals [38]–[41], but not in insects. Since insects are highly diverged compared with mammals, heterologous BAC-FISH will play a more important role in comparing insect genomes.

In contrast, BAC probes of *M. sexta* did not cross-hybridize onto *B. mori* chromosomes and *vice versa* under the conditions reported here (Fig. S2). This demonstrates that the overall sequence homology between these two representatives of different bombycoid families, Sphingidae and Bombycidae, is low, reflecting a relatively distant relationship. These observations are consistent with a recent molecular phylogenetic study which provides weak support for their generally accepted monophyletic origin [42], [43]. The evolutionary distance between the Sphingidae and Bombycidae has not been estimated. In Bombycoidea, the time of divergence between different lineages is difficult to calculate due to poorly understood phylogeny [43] and the lack of fossil records [44], which could be used to calibrate molecular clocks.

One of the most serious disadvantages of BAC-FISH analysis compared to recombination mapping is the limitation on the number of markers that can be applied in a single experiment, even using a multicolor BAC-FISH technique [26]. However, a reprobing protocol recently developed for lepidopteran material [30] enabled us to merge results obtained on the same chromosome from several experiments and greatly accelerated the delineation of the second lepidopteran karyotype. There remain the potential pitfalls that non–specific signals are misinterpreted as specific if a putative map position is consistent with expectation, or that specific signals are not identified due to unexpected chromosomal aberrations.

Traditional genetic linkage analysis remains essential to map loci encoding phenotypes, which is not possible with physical mapping by FISH. Nor can BAC-FISH mapping completely substitute for linkage analysis. Nevertheless, as discussed above, BAC-FISH has several advantages in initial physical characterization of the whole genome, which enables concentration of mapping efforts on a specific chromosome region by fine-scale linkage analysis. For instance, genetic markers can be designed from genes that are expected to be located in the region of interest on the basis of a comparative map which can be rapidly constructed by BAC-FISH.

### Conserved synteny of genes and conserved gene order in Lepidoptera

Besides a highly conserved gene order on a genome-wide scale and evidence of microsynteny, our detailed BAC-FISH comparison revealed only a few chromosomal rearrangements between the karyotypes of species representing two families of the ditrysian superfamily Bombycoidea, the Sphingidae (*M. sexta*; [27]) and the Bombycidae (*B. mori*; [42]), which have identical haploid chromosome numbers of n = 28 and a WZ/ZZ (female/male) sex-chromosome system [26]. All these rearrangements can be explained by eight chromosomal mutation events after the split of the two lineages of Bombycoidea from a common stem. We also found the nucleolar organizer region (NOR), composed of ribosomal DNA (rDNA), in different locations. In *B. mori*, a nucleolus formed by the NOR is associated with an interstitial region of chromosome 11 [25]. *M. sexta* also has a single NOR-bivalent with a nucleolus associated in a subterminal position [26]; however, the present study assigned the NOR to chromosome 28. A translocation of the NOR between chromosomes 11 and 28 could have occurred during fusion/fission events involving chromosomes 11 and 26 in a common ancestor (see Results). Similar transpositions of rDNA seem to be common in plants [45], [46] and also occur in insects [47], [48].

Lepidoptera are considered to have nearly holokinetic chromosomes [49], a chromosome type which occurs sporadically in distant phyla, e.g., in some monocot plants, hemipteran insects, mites, and nematodes [50]–[53]. Holokinetic chromosomes are characterized by the lack of a distinct primary constriction (the localized centromere), and their large kinetochore plates, also referred to as diffuse centromeres, cover a significant portion of the poleward surface of each sister chromatid [54], [55]. This structure makes chromosomal rearrangements more likely than in monocentric chromosomes because it reduces the risk of lethality caused by the formation of dicentric chromosomes and acentric chromosome fragments [50]. This property contrasts with the surprising stability of lepidopteran chromosomes revealed by comparative mapping of conserved synteny in this and other recent studies [13], [14]. Although higher resolution analysis provided by direct sequencing is likely to reveal additional chromosome changes (e.g., see [14], [56] ), our results demonstrate genome-wide collinearity of orthologous genes between *B. mori* and *M. sexta* and add to accumulating evidence for the overall stability of lepidopteran genomes despite different haploid chromosome numbers in certain clades [13], [14], [56]. Additional evidence of the extent of conserved synteny and gene order in more distantly related species is needed before drawing a more general conclusion about the extent of chromosome stability in Lepidoptera.

The isolated BAC clones presented in this report will be useful as primary resources for genome sequencing. Although EST projects are being extended to many other species and cDNA sequences are rapidly accumulating, most genomic sequences of higher eukaryotes are derived from limited model organisms. The BAC clones identified here can provide genome sequences of *M. sexta* confirmed to be orthologous with the corresponding *B. mori* sequences, which can be useful for detecting *cis*-elements such as enhancers and promoters as well as for gene annotation based on microsynteny [57]. BAC-FISH technology may also prove a useful tool for assembling short whole genome shotgun sequence fragments obtained by high throughput parallel sequencing methods from complex, repetitive genomes like those of Lepidoptera [7], [56].

Our study shows that BAC-FISH karyotyping and comparative mapping of genes is applicable to a wide range of species irrespective of conventional mapping efforts. Establishment of anchored physical maps using BAC-FISH will enable efficient acquisition of related target gene sequences from genomic libraries of the referenced species. Thus, BAC-FISH technology appears to be a promising tool for comparative genome analysis especially for widely diverged groups like insects for which there are currently few well-characterized genomes available, as well as for organisms having numerous and poorly distinguishable chromosomes. The finding reported here of a high degree of chromosomal conservation and establishment of preliminary reference maps to *B. mori* will facilitate gene annotation, gene discovery, and functional genomics in *M*. *sexta*, a leading laboratory model for insect biochemistry, physiology, neurobiology, development, and immunobiology. We have already applied this approach to several lepidopteran pests such as *Ostrinia nubilalis*, *Helicoverpa armigera*, and *Mamestra brassicae*, and succeeded in gaining preliminary results. Detailed synteny maps between these species and *B. mori* will be constructed in the near future, which will further facilitate comparative and functional genomics in Lepidoptera.

## Materials and Methods

### Selection of genes for comparative mapping between *Manduca sexta* and *Bombyx mori*

Sequences of *M. sexta* genes and ESTs were obtained from public databases or ButterflyBase [58] and used as queries for TBLASTN searches against the whole genome shotgun (WGS) [28], [29] using the BLAST tool associated with a database of the silkworm genome, Kaikoblast (http://kaikoblast.dna.affrc.go.jp/). *M. sexta* genes showing significant similarity to putative single-copy *B. mori* genome sequences were selected and checked for previous localization of their *B. mori* orthologs [12]. If not, we performed PCR-based linkage analysis of unmapped orthologs with 86 newly designed primers for sequence-tagged sites (STS) (Table S1) using 166 F_2_ individuals of *B. mori* from a single pair-mating of a strain C108 female by a strain p50 male, as reported previously [59].

### Isolation of BAC clones

BAC clones used as FISH probes were isolated from a BAC library of *M. sexta* as described previously [26]. Briefly, primer sets based on *M. sexta* genes were designed to avoid including putative exon-intron junctions predicted from the alignment of cDNA sequences of *M. sexta* with genome sequences of *B. mori* (Table S4). The primers were used for PCR-based screening of 18,400 clones of an *M. sexta Eco* RI BAC library [23] (384-well plate no.'s 1–47, 49) obtained from the GENE*finder* Genomic Resources (Texas A&M University, College Station, TX, USA).

Screening was performed in three steps as described previously [60]. The first screening was performed against DNA pools derived from 48 plates, using a mixture of 384 BAC-DNAs for each plate. The second screening was carried out only in positive plates by amplifying PCR products consistent with those generated from the genomic DNA template. DNA pools for 24 columns and 16 rows, each composed of mixtures of BAC-DNAs located in the same column or row, were used as templates. Finally, candidates identified by the preceding two steps were individually amplified to confirm the presence of target sequences. Clones containing the *Broad-complex*, E75, ephrin receptor, fasciclin II and nitric oxide synthase genes were first screened by *in situ* hybridization using high-density replica filters [23], and then confirmed by PCR amplification.

### BAC-FISH mapping

We followed essentially the procedure described previously [26] with slight modifications in multi-color BAC-FISH. Chromosome spreads were prepared from pachytene spermatocytes (and if needed from pachytene oocytes) of *M. sexta* and *A. convolvuli* larvae. BAC-DNA was extracted with a Plasmid Midi kit (QIAGEN GmbH, Hilden, Germany) and labeled with a fluorochrome using a Nick Translation System (Invitrogen, Carlsbad, CA, USA), with one of four fluorochromes, which were discriminated with different filter sets. These were Green-dUTP, Orange-dUTP, and Red-dUTP (Abbott Molecular Inc., Des Plaines, IL, USA), and Cy5-dUTP (GE Healthcare UK, Buckinghamshire, UK). For BAC-FISH, a cocktail of BAC-DNA probes was hybridized to male chromosome preparations. For genomic *in situ* hybridization (GISH), female genomic DNA labeled with Green-dUTP using the Nick Translation System was hybridized to the female chromosome preparation as described previously [61]. In some experiments, we used a protocol for reprobing pachytene nuclei processed by multi-color BAC-FISH recently developed for Lepidoptera [30]. Further details about BAC-FISH mapping are available in our previous papers [12], [25]. In all, we analyzed 2,218 chromosome spreads from 109 preparations of *M. sexta* and 60 chromosome spreads from 5 preparations of *A. convolvuli*.

In *M. sexta*, we estimated the relative position of the BAC-FISH signals using Image J 1.42 software (http://rsbweb.nih.gov/ij/download.html). The length of pachytene bivalents varies considerably due to differences in condensation between nuclei and spreading procedure. Therefore, we selected 5 representative bivalents for each chromosome, except for chromosomes 14 (n = 4) and 16 (n = 3), and calculated the median distance of hybridization signals relative to the proximal end of the corresponding *B. mori* linkage map (Fig. S2).

## Supporting Information

Figure S1 A physical map of *Manduca sexta* based on the relative position of BAC-FISH signals. The numbers on the left side of bars show the median distance (%) of hybridization signals relative to the proximal end of the corresponding *B. mori* linkage map, calculated from 3–5 representative pachytene bivalents. The numbers on the right side are accession numbers of the orthologous genes used for selecting BAC clones (see Table S5 for details). Chromosome numbers (Z = 1) are listed above the bars.
(0.07 MB PDF)

Figure S2 Cross-hybridization of BAC probes between *Manduca sexta* and *Bombyx mori*. Shown are *M. sexta* BAC probes hybridized to male pachytene bivalents of *B. mori* (a–c) and *B. mori* probes to male pachytene bivalents of *M. sexta* (g–i). Note the absence of hybridization signals on the bivalents; arrows indicate some conjugates of fluorochrome-labeled probes outside bivalents. Control hybridizations of the probes to bivalents of the respective species are shown in d–f (*M. sexta* ) and j-l (*B. mori* ); note specific hybridization signals on the corresponding bivalent(s). Codes of *M. sexta* BAC probes tested: 15J11 (cyan), 22D05 (red), 34M13 (yellow), and 18A03 (green) (Z chromosome, see a and d); 40I18 (cyan), 39P21 (red), 49K12 (yellow), and 43G05 (green) (chromosome 5, see b and e); 39L03 (cyan), 02I22 (red), 10O14 (yellow), and 30J18 (green) (chromosome 15, see c and f). Codes of *B. mori* BAC probes tested: 9B9D (cyan), 9C6H (red), 9A5H (yellow), and 5I3F (green) (Z chromosome, see g and j); 4B2A (cyan), 6F8D (red), 1B9C (yellow), and 9A12 (green) (chromosome 5, see h and k); 2E9E (red), 7D3E (green) (chromosome 15), 10L7B (yellow) (chromosome 20), and 8C2C (cyan) (chromosome 23; see i and l). For BAC codes, see Table S5 for *M. sexta* and Yasukochi et al. (2006) for *B. mori*. Bar represents 5 µm for a and 10 µm for b-l.
(2.07 MB TIF)

Table S1 Polymorphic STS primers of *Bombyx mori* orthologous genes designed for these experiments.
(0.11 MB DOC)

Table S2 Mapping of *Bombyx mori* orthologs of *Manduca sexta* genes. Genes and ESTs of *M. sexta* are sorted into *B. mori* LGs to which orthologs belong. Ref. 1. Yasukochi, Y, Ashakumary L, Baba K, Yoshido A, Sahara K. (2006) Genetics 173:1319–1328. 2. Miao XX, Xub SJ, Li MH, Li MW, Huang, JH et al. (2005) Proc Natl Acad Sci USA 102: 16303–16308.
(0.39 MB DOC)

Table S3 Gene anchors in *Manduca sexta* BACs. Requests for clones or the library should be sent to H.B.Z. Notes: Clones positive for multiple genes are underlined.
(0.16 MB DOC)

Table S4 STS primers used for PCR screening of the *Manduca sexta* BAC library.
(0.16 MB DOC)

Table S5 Summary of BAC-FISH analysis. * G, O, R, C5: BAC probes labeled with Green-dUTP, Orange-dUTP, Red-dUTP and Cy5-dUTP and pseudocolored for green, yellow, red and cyan if not specifically mentioned; - represents BAC not used in Fig. 1 but in Fig. 2. ** According to Kaikobase (http://kaikoblast.dna.affrc.go.jp/) a The upstream region was misintegrated to chr. 13 in Kaikobase. b The upstream region was mistakenly integrated to chr. 3 in Kaikobase.
(0.25 MB DOC)
