# Supplementary figures and images for: Extensive Conserved Synteny of Genes between the Karyotypes of Manduca sexta and Bombyx mori Revealed by BAC-FISH Mapping
Source: PLoS One. 2009 Oct 15;4(10):e7465. doi: 10.1371/journal.pone.0007465 (PMC2759293; doi:10.1371/journal.pone.0007465)

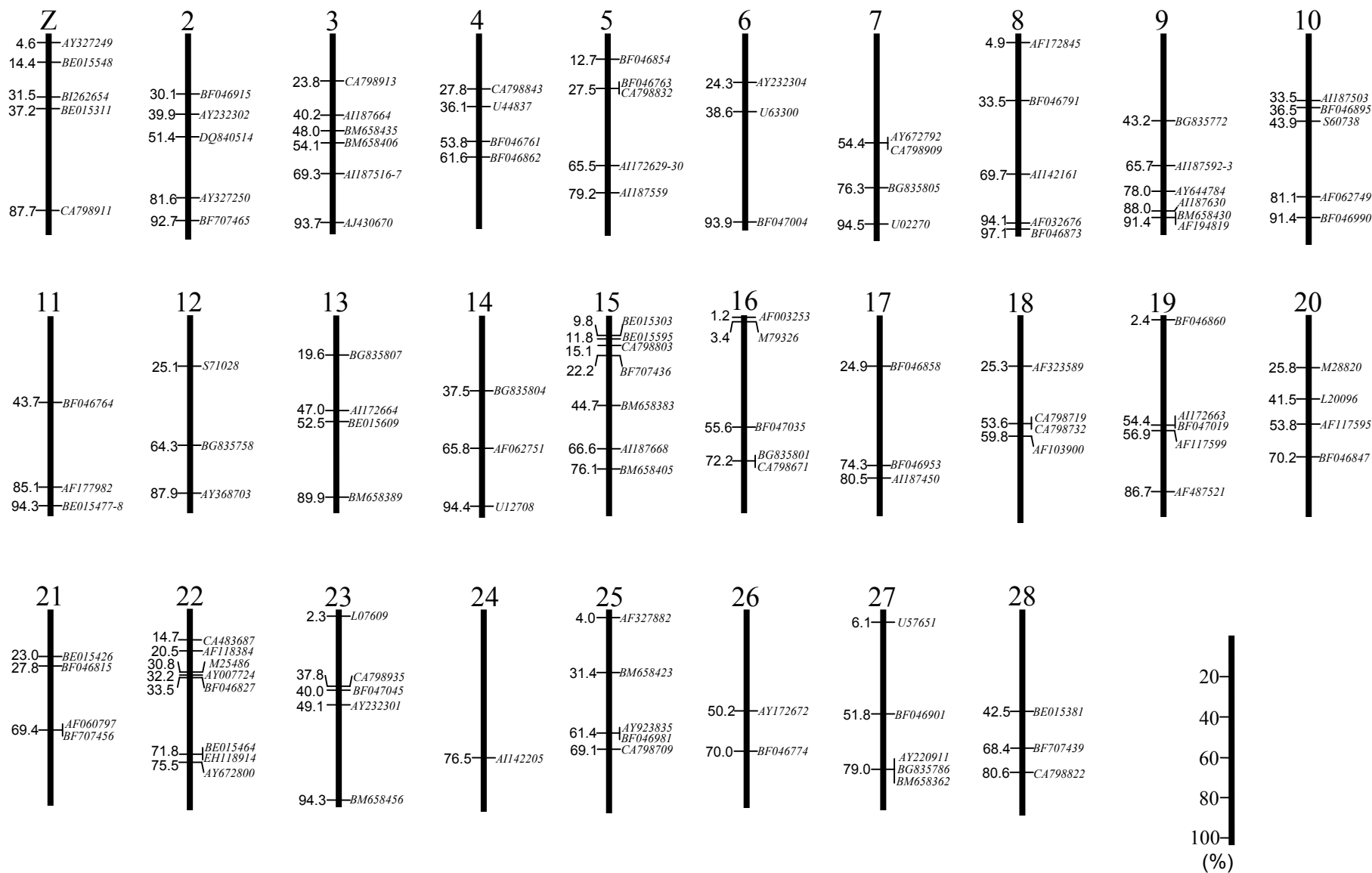

Supplement: Figure S1 — A physical map of Manduca sexta based on the relative position of BAC-FISH signals. The numbers on the left side of bars show the median distance (%) of hybridization signals relative to the proximal end of the corresponding B. mori linkage map, calculated from 3–5 representative pachytene bivalents. The numbers on the right side are accession numbers of the orthologous genes used for selecting BAC clones (see Table S5 for details). Chromosome numbers (Z = 1) are listed above the bars. (0.07 MB PDF) [file pone.0007465.s001.pdf]

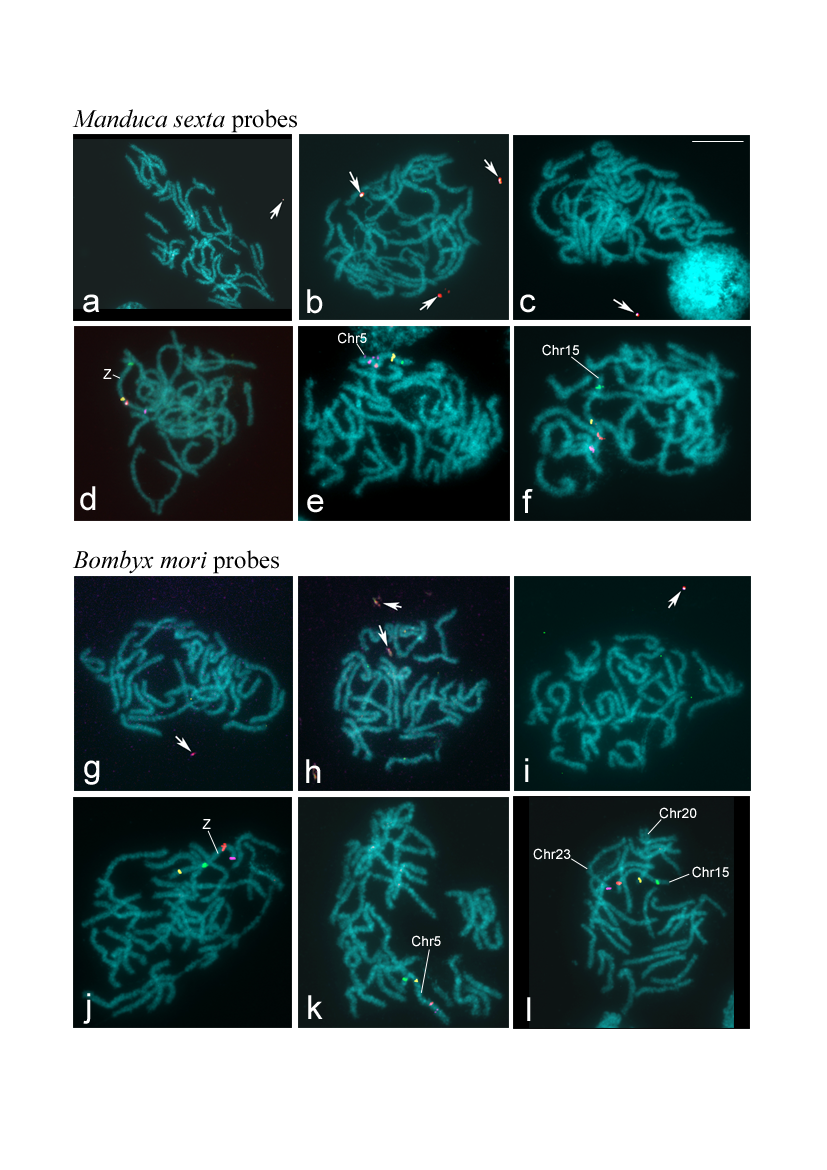

Supplement: Figure S2 — Cross-hybridization of BAC probes between Manduca sexta and Bombyx mori. Shown are M. sexta BAC probes hybridized to male pachytene bivalents of B. mori (a–c) and B. mori probes to male pachytene bivalents of M. sexta (g–i). Note the absence of hybridization signals on the bivalents; arrows indicate some conjugates of fluorochrome-labeled probes outside bivalents. Control hybridizations of the probes to bivalents of the respective species are shown in d–f (M. sexta ) and j-l (B. mori ); note specific hybridization signals on the corresponding bivalent(s). Codes of M. sexta BAC probes tested: 15J11 (cyan), 22D05 (red), 34M13 (yellow), and 18A03 (green) (Z chromosome, see a and d); 40I18 (cyan), 39P21 (red), 49K12 (yellow), and 43G05 (green) (chromosome 5, see b and e); 39L03 (cyan), 02I22 (red), 10O14 (yellow), and 30J18 (green) (chromosome 15, see c and f). Codes of B. mori BAC probes tested: 9B9D (cyan), 9C6H (red), 9A5H (yellow), and 5I3F (green) (Z chromosome, see g and j); 4B2A (cyan), 6F8D (red), 1B9C (yellow), and 9A12 (green) (chromosome 5, see h and k); 2E9E (red), 7D3E (green) (chromosome 15), 10L7B (yellow) (chromosome 20), and 8C2C (cyan) (chromosome 23; see i and l). For BAC codes, see Table S5 for M. sexta and Yasukochi et al. (2006) for B. mori. Bar represents 5 µm for a and 10 µm for b-l. (2.07 MB TIF) [file pone.0007465.s002.tif]
