# Supplementary material for: Extensive Conserved Synteny of Genes between the Karyotypes of Manduca sexta and Bombyx mori Revealed by BAC-FISH Mapping
Source: PLoS One. 2009 Oct 15;4(10):e7465. doi: 10.1371/journal.pone.0007465 (PMC2759293; doi:10.1371/journal.pone.0007465)
Supplement: Table S1 — Polymorphic STS primers of Bombyx mori orthologous genes designed for these experiments. (0.11 MB DOC) [file pone.0007465.s003.doc]

| *M. sexta* | *B. mori* ortholog | Forward primer sequence | Reverse primer sequence | Product size |
| --- | --- | --- | --- | --- |
| BE015548 | BP184063 | TGTGATTGAGATTCGTTTT | CCATTATGCTGAGGTGAGA | 482 |
| BI262545 | CK516660 | GTAATCTCCAACCAAAATC | AATCATAGCGGCACACCTC | 424 |
| CA798911 | BP115945 | TCTATGACCCTGATTCTGG | CTGTTTTTGACCTCCTGAC | 449 |
| BE015311 | DC542913 | TAGTGGTGAGATGATGAGG | GGTTTGGTGTTCCGATGAC | 662 |
| BF047022 | BP125823 | GGTCTGAAGATGGAATGTA | CCTTTGAATGATGTATGTA | 543 |
| AY585211 | BY914831 | ATAGAGTGGGAACAACAGC | CAAACAGTGGAGGTGACAT | 583 |
| AI187655 |  | TTGGCAGATAGGCGATAC | AGATTTACTGTGGGAGAG | 655 |
| AI187662 | BP182373 | TTTATCATTTTGTCGTTTGT | GCTTCTGCTATTCTTTTTCT | 512 |
| BF047007 | CK534146 | CAGAAAACAGACAACTATGG | TAAAACGAAACAAAGAAAGG | 387 |
| BF046854 | BP182348 | TGCGTAATACTAATCAAGC | TGGAAAACATCAAACAAAG | 403 |
| CA483695 |  | AATAATCCCAACAATCAAC | ATACCCGTAAATCAAACAA | 667 |
| BF047063 | BB991071 | TCAACAAACTTCCCTCCAT | GTCTTATAGTCGCCCTCAG | 367 |
| AI172629, AI172630 | BP183504 | CCAGCCTCCAATCATCACC | TCAGTTCAGTTCAAAGTCA | 543 |
| AI187559 | BW998437 | TATGTCTGATGCTGGAAAA | CTTGCTGGCTCTGTGGTCT | 680 |
| CA798805 | CK501723 | TGAAATGTTGTGAATGTGA | CGATGACGGTAATGTTGGA | 563 |
| BF046767 |  | ACAAAGATAGCAGCATAAA | GGAGTAAGCACACAGAAAA | 266 |
| AI172623 | BY914451 | CCGTCTCTTTCGTTTCATA | TGTGTTTTCTTTTTCTTTC | 376 |
| CA798909 | DQ311339 | TAGAAATAACGAGCACTGA | ACAAAGGCAAAAGGCACAA | 428 |
| AF413065 | DQ311310 | GATACGAGCAACAAAACAC | TATTCCACGACTAAGCAAA | 506 |
| AF393501 | DQ311309 | GTTCTCCTTCTGCTCATCTG | TTTTATCGTGTTCGTCGTCT | 371 |
| AI142176 | DQ311227 | GACCCACCAGGACGATAA | CACTTTGCCAGCCACTAC | 394 |
| AF172845 | AV401968 | AACTTCTTATCGCACCTTA | CGCTCACCACTTCGTCTG | 347 |
| BF046791 | BY923515 | ACCAAGAACAACAACAGCAC | ACCAACTAAATCAGCCACAG | 507 |
| AI142161 |  | GATTTGTGTTTTGTTGTGA | AGTTTTATCCGAAGTTGTA | 628 |
| BM658364 | CK556527 | ATTTAGCATTCCGAGAGCA | CGCATCCAGTCCTTACCAG | 697 |
| CA798819 | BY916638, BY934883 | AGAGGTTATTAGTCAGGTCA | GATGGTCCCGAGAAGAAGAT | 652 |
| BF046873 | DC532116 | AAAAATCAAACTAATCAAAAT | CTCCTCAGAAAGCCAATAAT | 434 |
| AI187630 | BY918457 | TCACATTTAGTCCGATTATT | GAAAACGATTGCCTCTTGG | 840 |
| AY644784 | CN375578 | TTTGTTTAGTGAGTAGTGC | GTTCTTAGACCAGGACGAC | 714 |
| BG835802 | CK561386 | GCCACTACCACCCTTCTAT | GCTCGGATGTTGACCTTCT | 540 |
| AI187592, AI187593 | CK500660 | TTGCTAACTACACTAAGACA | ATGAATACAAAACAGATGG | 637 |
| BM658398 | AV398545 | CGATAGTCAGGAGTGTAAGG | ATAGAAAAGGGAAGAAAGAA | 612 |
| AF053131 | AY297159 | ATCGTTTCCTGCTCGTATTC | ATTGCTGTTATCTTTCCATT | 597 |
| BI262649 | CK560761 | GGAGAGGGGAGCGTCGTAA | ACATCAGGTCAGTATTCGT | 652 |
| BM658385 | CK533914 | CAGGACAGGTGGACGGGTAA | ATGAGGGAAATGGAAGGACT | 792 |
| AI187506, AI187505 |  | ACACCAGACAACACGAGGAG | CAGAACGAAGGGCATTAGTA | 478 |
| BM658404 | CK562538 | AAATGAATGGTGGGACAGA | ACTTGGCAGTTGATAATGA | 663 |
| BF046774 | BJ985383 | AAGCAATAATAAAATGGAT | CTGGTGGATGATAGGAACG | 965 |
| AI187536 | BP117568 | CTTGTCTGCGAGGGGTCAC | CAGGCGGCTTTGTAGGTTA | 690 |
| AY368703 | S77548 | AAAAGTGGGGGAGAAAAAC | ATCAAAACAACAGAAGTAAT | 552 |
| BF707453 | AV403631 | TTCAAGTGAGTGTAACGAC | AAATAAATAACCCGACCTG | 638 |
| AI187470 | BY921003 | TACCTCTACCCTCCATAATC | AAAACGCTCTCACGAAATCT | 446 |
| BF046807 | AY769315 | CGATAATCTAATGACAGCA | TGACAGGAACCCACTCTTT | 576 |
| BF047031 | CK493808 | TGTCAGGGTGTATTTTATGC | ATTTCAAGAAGATGGGTGTT | 504 |
| U12708 | AY769316 | AGCCATCGCCGTCCCTCTT | AACTCCATCTCCAACAAAC | 398 |
| CA483669 | CK488562 | TTGTGTGGAATGGGTGTGT | AAAACGACTTAGGTGGATA | 346 |
| BG835801, CA798671 | DQ443264 | ACATTTTTGTCCTTTCATT | ACTCCGCTTGGGTATTTCA | 340 |
| U75307 | DQ109670 | TTCGTGGACCCGTAATCTA | GTCACCTCTTCAACCTTCA | 676 |
| CA798787 | DQ311380 | GATACATTGGTGGTTTCAGTG | AATAATCTTCAGTTGGGTTTT | 821 |
| AI187574 | BB985260 | GCTTCTCATTCTCAGGGTAA | ATTGGATGTGGTTCAGGATT | 887 |
| BF046977 | DQ443365 | CATTTGATTTGTTGTGGT | TTGCGTTTTAGCGTTTCC | 746 |
| AI187450 | DQ497202 | CAGAGGATTTCGTAGAGATT | TCGTGGGGTAGTATTCGTTG | 412 |
| BF046858 | AY769299 | GGTGGATTCGTGCCTGGTCT | TCTGATTCAAAGTGGTTCGT | 417 |
| AI142209 | CK561983 | TTTTGATTTCTTTTTGACTG | ACTGTTCCACTCGGTTGATT | 558 |
| CA798732, CA798719 | BY914105 | TATAGCCGCCGTTTTAGG | AATCATAGACCGACTTCA | 463 |
| AF103900 | BP114941 | AACTTGTCTTCACTGCTTAG | CTTACGGGATTTTCTTCTTT | 993 |
| AF487521 | BP125399 | CACCAGACCCCACGAGAAG | CGAGAACGGATACCAACCT | 630 |
| AF117599 | AJ973405 | AACAGGTACTTTTCTCAGG | GTCTTTGTATCTCGCTTGG | 786 |
| BI262622 | DQ311275 | CGTCGTATAGAAACATTAGC | AAAACTGAGGAAGCCATTGA | 792 |
| BF046860 | DQ443396 | CCTCTTTCTCGTTTCCTGT | AATGAGTCGTTCGGTTTTA | 430 |
| AF117595 | DQ443423 | TACCTTTGCCTTTCCAGTTC | ATTTCGCTCTTTCCCCTACA | 592 |
| BF046847 | DQ443282 | GCTTCTACAACCTACAATC | TGAATACTAACGCATCTCG | 1011 |
| U17344 | CK544274 | GGGCTCGTGCTCCTTGTTC | TGTTTCGGTATTTCTTTTA | 991 |
| BF707456 | DQ311253 | TGTCCGAGCACTTTATCTAC | CGTTGTCATTTTCATTTTCC | 612 |
| BF046827 | BP183480 | GCCTACGGATACCAAACTC | AACCCTCTCTTCTTCTGTG | 505 |
| BE015557 | CK510334 | ATGCCAGAAAAGTAATGCT | GCCTGAAGTTGCTAAGAAA | 525 |
| AF117587 |  | CCACCAAAACGAAAGGCTAC | TCAGATAAGACGCTCACAAC | 626 |
| CA798823 | BY939117 | CATTTTTCGTTTTGTTTGTT | GAGGTACTGATTGTTTGTGG | 831 |
| BM658405 | BY938896 | TTTGAGCAGCATCCCTTTAC | TCATTATCAGCAGACTTGTT | 744 |
| CA798873 | BY924190 | TAAAGATACGAGCATAAGC | CGAATAAGCCACAGATAGT | 471 |
| CA483685 | DQ311432 | CTTTCTTTGTTTTTCTTCCAG | TTATGTGTCGGTTTGTGTTTC | 700 |
| BF047045 | BW999258 | TTTTCTTGTTTGAGGGTGTT | CTAAAAGCTCAAGTAAATCG | 536 |
| BI262571 | BJ985900 | CTTCGTTTTTAGCCTTGTTC | ATTAGTGTCATTTTCCTTCC | 332 |
| BE015464, EH118914 | DQ443196 | ATGATTTTGACCCCTCTTT | GTTTTCTTTTTCCCTACCT | 1062 |
| L47123 | DQ443250 | ATGCCTTCAGTTCGTTCG | AGTTTGTTTGTTCTCGTT | 654 |
| BF046892 | BJ985752 | GGTGTGGTTGGTATTATTTG | ATGAGCCTTTATGATGATTT | 271 |
| BE015326 | BY924492 | AAAAGGAAAAGGAAAAGCA | TCAGCATAGGTAGGAAAAT | 498 |
| BM658423 | DQ311171 | AATGACTTCGCTTGCTAACT | TAATGACCCTTTGCCACTTG | 394 |
| BF046995 | AV398721 | ACACAATGGTTACGACTTTT | TGATTATTCCGAGACTGACT | 188 |
| BF046981 | BY914040 | CCTTCCTTGCCTTCATCCT | CATAGTCGTTTTCCTTTCG | 762 |
| BF046915 | BP123427 | CAAAGGAAGGTCGTATCAAA | GAGAAGTCAGGAGTGGCTAA | 995 |
| DQ840514 | AB255163 | GCTCGTACACCTAACTATG | TCCTGCTCCTCCAAAGAAT | 291 |
| CA798822 |  | ATACGACATTTAGAGATAGC | GATTTGGAAGTTACAGAAGA | 274 |
| AY672781 |  | AACAATGGGAGACTTACCTA | ATCGTGGAACAAAACTGAAT | 800 |
| BE015381 | CK530917 | CCTTCGCCTTTCTCAATG | CCTTCCCAAGCCAATACT | 656 |
| AJ249388 | DQ311199 | TATTCAGTCATTTTCACCAC | GAGTTTCCAGTTCTTCTATT | 706 |
