# Supplementary material for: Extensive Conserved Synteny of Genes between the Karyotypes of Manduca sexta and Bombyx mori Revealed by BAC-FISH Mapping
Source: PLoS One. 2009 Oct 15;4(10):e7465. doi: 10.1371/journal.pone.0007465 (PMC2759293; doi:10.1371/journal.pone.0007465)
Supplement: Table S2 — Mapping of Bombyx mori orthologs of Manduca sexta genes. Genes and ESTs of M. sexta are sorted into B. mori LGs to which orthologs belong. Ref. 1. Yasukochi, Y, Ashakumary L, Baba K, Yoshido A, Sahara K. (2006) Genetics 173:1319–1328. 2. Miao XX, Xub SJ, Li MH, Li MW, Huang, JH et al. (2005) Proc Natl Acad Sci USA 102: 16303–16308. (0.39 MB DOC) [file pone.0007465.s004.doc]

| *M. sexta* gene | | *B. mori* ortholog | | | | |
| --- | --- | --- | --- | --- | --- | --- |
| Gene | EST | Gene | EST | LG^1^ | Contig code^1^ | Mapping method |
|  | BE015508 | AY734490 |  | 1(Z) |  | FISH |
| AY327249 |  |  |  | 1(Z) | 01_1 | located on mapped contigs |
|  | BE015548 |  | BP184063 | 1(Z) |  | directly mapped |
|  | BI262654 |  |  | 1(Z) | 01_7 | located on mapped contigs |
|  | CA483713 |  |  | 1(Z) | 01_7 | located on mapped contigs |
|  | BI262545 |  | CK516660 | 1(Z) |  | directly mapped |
|  | CA798915 |  | CK536289 | 1(Z) |  | located on mapped contigs |
| AF288089 |  | D10953 |  | 1(Z) | 01_10 | located on mapped contigs |
|  | AI142156 | AB098537 |  | 1(Z) | 01_11 | directly mapped |
|  | CA798911 |  | BP115945 | 1(Z) | 01_12 | directly mapped |
|  | BE015311 |  | DC542913 | 1(Z) | 01_21 | directly mapped |
|  | BF047022 |  | BP125823 | 2 | 02_1 | directly mapped |
|  | CA798718 |  | BB990930 | 2 | 02_2 | located on mapped contigs |
|  | CA483683 | AY769292 |  | 2 |  | located on mapped contigs |
| AY616435 |  | EF362786 |  | 2 | 02_6 | located on mapped contigs |
|  | CA798800 |  | AU005437 | 2 |  | located on mapped contigs |
| AY327250 |  |  | BY922552 | 2 | 02_7 | located on mapped contigs |
| M23438 |  |  | BB990670 | 2 | 02_7 | located on mapped contigs |
| AF117600 |  |  | CK555707 | 2 | 02_8 | located on mapped contigs |
| AY585211 |  |  | BY914831 | 2 | 02_8 | directly mapped |
|  | BF707465 |  | BY935853 | 2 | 02_10 | located on mapped contigs |
|  | AI187655 |  |  | 2 |  | directly mapped |
|  | BF047041 | AY578154 |  | 2 | 02_11 | directly mapped |
|  | CA798943 |  | CK513631 | 2 | 02_11 | located on mapped contigs |
|  | BE015498 | AY769341 |  | 3 |  | directly mapped^2^ |
|  | BF047053 | AB190802 |  | 3 | 03_3 | directly mapped |
|  | CA798913 |  | BP120290 | 3 |  | FISH |
|  | AI187664 |  | CK517579 | 3 | 03_5 | located on mapped contigs |
|  | BM658435 | AY769283 |  | 3 | 03_9 | directly mapped |
| AJ249389 |  | DQ443403 |  | 3 | 03_10 | located on mapped contigs |
|  | BM658406 | AY769280 |  | 3 | 03_10 | directly mapped |
|  | AI142211 |  | BP120129 | 3 | 03_10 | located on mapped contigs |
|  | AI187662 |  | BP182373 | 3 | 03_11 | directly mapped |
|  | AI187516, AI187517 |  | DC561789, DC573084 | 3 | 03_13 | located on mapped contigs |
|  | BF046867 | D13339 |  | 3 | 03_13 | located on mapped contigs |
|  | BI262591 |  | BB990671 | 3 | 03_14 | located on mapped contigs |
| AJ430670 |  | AY363308 |  | 3 | 03_18 | located on mapped contigs |
|  | CA798774 |  | BP116713 | 4 |  | located on mapped contigs |
|  | CA798843 |  | CK538412 | 4 | 04_2 | located on mapped contigs |
| U44837 |  | AB182582 |  | 4 | 04_6 | directly mapped |
|  | BF047007 |  | CK534146 | 4 |  | directly mapped |
|  | BF046761 | AY579746 |  | 4 | 04_11 | directly mapped |
|  | CA798744, EH118875 | AB024901 |  | 4 | 04_11 | located on mapped contigs |
| AY062175 |  |  | BP116062 | 4 | 04_12 |  |
|  | BF046957 | DQ118523 |  | 4 | 04_12 | directly mapped |
| U03909 |  | AF372836 |  | 4 | 04_13 | located on mapped contigs |
|  | BE015617 | AY769334 |  | 4 | 04_13 | located on mapped contigs |
|  | BF046862 | DQ150250 |  | 4 | 04_14 | located on mapped contigs |
| AF060794 |  | DQ311347 |  | 5 | 05_1 | located on mapped contigs |
|  | BF046854 |  | BP182348 | 5 |  | directly mapped |
|  | CA798754 |  | CK563184 | 5 | 05_3 | located on mapped contigs |
|  | BF046763, CA798832 |  | BP181302 | 5 | 05_5 | located on mapped contigs |
|  | CA798889, EH118892 | DQ311355 |  | 5 |  | FISH |
|  | CA483695 |  |  | 5 |  | directly mapped |
|  | BF047063 |  | BB991071 | 5 |  | directly mapped |
|  | BF707457 | AY769282 |  | 5 | 05_8 | located on mapped contigs |
|  | BI262575 |  | CK507707 | 5 | 05_8 | located on mapped contigs |
|  | AI187543, EH118452, BE015294 | DQ311235 |  | 5 | 05_11 | located on mapped contigs |
|  | CA798728 |  | BP115472 | 5 | 05_13 | located on mapped contigs |
|  | AI172629, AI172630 |  | BP183504 | 5 |  | directly mapped |
|  | BF047058 | AY769289 |  | 5 | 05_18 | directly mapped |
|  | CA798826 |  | AV403293 | 5 | 05_18 | located on mapped contigs |
|  | AI187559 |  | BW998437 | 5 |  | directly mapped |
| AF234571 |  | D13338 |  | 5 |  | FISH |
| AY232304 |  | AF005384 |  | 6 | 06_1 | located on mapped contigs |
| BE015347, BE015348 | MSC00405 |  |  | 6 | 06_1 | located on mapped contigs |
| CA798805 | MSC01336 |  | CK501723 | 6 | 06_1 | directly mapped |
| BF046767 | MSC00028 |  |  | 6 | 06_6 | directly mapped |
| AI172623 | MSC00096 |  | BY914451 | 6 | 06_6 | directly mapped |
| BF046804 | MSC00611 |  | AU004425 | 6 | 06_6 | located on mapped contigs |
| M26922 |  | D10135 |  | 6 |  | located on mapped contigs |
| AF117596 |  | AJ006502 |  | 6 | 06_13 | located on mapped contigs |
| S77989 |  | X62620 |  | 6 | 06_16 | located on mapped contigs |
| U63300 |  | X62618 |  | 6 | 06_16 | located on mapped contigs |
| U63301 |  | D16684 |  | 6 | 06_16 | located on mapped contigs |
|  | BF047065 | AY769285 |  | 6 | 06_18 | directly mapped |
|  | BF047004 | BN000406 |  | 6 | 06_19 | located on mapped contigs |
| AY094541 |  | AB041508 |  | 6 | 06_21 | directly mapped |
| AY672792 |  | AB073673 |  | 7 | 07_3 | directly mapped |
|  | CA798909 | DQ311339 |  | 7 | 07_3 | directly mapped |
|  | BG835805 | AY192575 |  | 7 | 07_5 | located on mapped contigs |
| AJ863121 |  | AB030498 |  | 7 | 07_8 | located on mapped contigs |
|  | CA483678, BG835756 | AY769342 |  | 7 | 07_10 | located on mapped contigs |
| U02270 |  | AB052914 |  | 7 | 07_11 | directly mapped |
|  | AF413065 | DQ311310 |  | 7 |  | directly mapped |
| AF393501 |  | DQ311309 |  | 7 |  | directly mapped |
|  | AI142176 | DQ311227 |  | 7 | 07_17 | directly mapped |
| AF172845 |  |  | AV401968 | 8 | 08_1 | directly mapped |
|  | BF046791 |  | BY923515 | 8 | 08_11 | directly mapped |
|  | BM658424 | DQ311422 |  | 8 | 08_15 | located on mapped contigs |
|  | CA483679 | AF317420 |  | 8 | 08_15 | directly mapped |
|  | AI142161 |  |  | 8 |  | directly mapped |
|  | BM658364 |  | CK556527 | 8 |  | directly mapped |
|  | CA798819 |  | BY916638, BY934883 | 8 |  | directly mapped |
| AF032676 |  | AB113088 |  | 8 | 08_18 | directly mapped |
|  | BF046873 |  | DC532116 | 8 |  | directly mapped |
|  | BM658430 |  | BP178402 | 9 | 09_2 | located on mapped contigs |
| AF194819 |  | AB084922 |  | 9 | 09_2 | directly mapped |
|  | AI187630 |  | BY918457 | 9 | 09_5 | directly mapped |
| AY644784 |  |  | CN375578 | 9 |  | directly mapped |
|  | BG835802 |  | CK561386 | 9 |  | directly mapped |
|  | CA798930 | AB206402 |  | 9 | 09_7 | directly mapped |
|  | BE015486, CA483690 | AY706956 |  | 9 | 09_8 | located on mapped contigs |
|  | AI187592, AI187593 |  | CK500660 | 9 |  | directly mapped |
|  | BG835772 | AY769294 |  | 9 | 09_13 | located on mapped contigs |
|  | BM658398 |  | AV398545 | 9 |  | directly mapped |
| X97877 |  | AB011497 |  | 9 | 09_19 | directly mapped |
| AF053131 |  | AY297159 |  | 10 |  | directly mapped |
|  | CA798956 | D29738 |  | 10 | 10_4 | directly mapped |
| AY135186 |  |  | BY916169 | 10 | 10_8 | located on mapped contigs |
|  | AI142167 | DQ311166 |  | 10 | 10_8 | located on mapped contigs |
|  | AI187503 |  |  | 10 | 10_7, 10_10 | located on mapped contigs |
| U19812 |  | D87118 |  | 10 | 10_9 | located on mapped contigs |
|  | BF046895 |  | BP183438 | 10 | 10_9 | located on mapped contigs |
| FJ530954 |  | AB003035 |  | 10 | 10_11 | directly mapped |
| S60738 |  | AB024904 |  | 10 | 10_13 | located on mapped contigs |
|  | BM658431 | DQ311354 |  | 10 | 10_13 | located on mapped contigs |
| DQ840516 |  | X95604 |  | 10 | 10_14 | located on mapped contigs |
| AF117572 |  |  | AV405651 | 10 | 10_15 | located on mapped contigs |
| AF062749 |  | AB017521 |  | 10 | 10_18 | located on mapped contigs |
| L11449 |  | AF246695 |  | 10 | 10_24 | directly mapped |
|  | BF046990 | DQ086424 |  | 10 | 10_24 | directly mapped |
|  | BI262649 |  | CK560761 | 10 |  | directly mapped |
|  | AI187640 |  | CK560305 | 11 |  | located on mapped contigs |
|  | CA798942 |  | BY936521 | 11 | 11_3 | located on mapped contigs |
|  | BM658385 |  | CK533914 | 11 | 11_7 | directly mapped |
|  | BF046764 | AY769284 |  | 11 | 11_8 | located on mapped contigs |
|  | AI172747 |  | BP114835 | 11 | 11_10 | located on mapped contigs |
|  | BF046844 | AY769287 |  | 11 | 11_10 | directly mapped |
|  | AI187506, AI187505 |  |  | 11 | 11_11 | directly mapped |
| L78081 |  | AB047925 |  | 11 | 11_11 | directly mapped |
|  | BM658404 |  | CK562538 | 11 |  | directly mapped |
|  | CA798667 |  | CK487989 | 11 | 11_14 | located on mapped contigs |
| AF177982 |  | AB026441 |  | 11 | 11_15 | located on mapped contigs |
|  | BF046751 | DQ443337 |  | 11 | 11_15 | located on mapped contigs |
|  | BE015478, BE015477 |  | AU002885 | 11 | 11_17 | located on mapped contigs |
|  | AI234460, AI234461 | DQ443122 |  | 11 | 11_17 | located on mapped contigs |
|  | AI187597, BF046919 | AY769271 |  | 11 | 11_20 | directly mapped |
| AY172672 |  | D16230 |  | 11 | 11_21 | located on mapped contigs |
|  | BF046774 |  | BJ985383 | 11 |  | directly mapped |
| S71028 |  | L37416 |  | 12 | 12_2 | directly mapped |
| AF176015 |  | AF176014 |  | 12 | 12_7 | located on mapped contigs |
|  | AI187536 |  | BP117568 | 12 |  | directly mapped |
|  | BG835758 | AY769277 |  | 12 | 12_8 | located on mapped contigs |
| AY368703 |  | S77548 |  | 12 |  | directly mapped |
|  | BF707453 |  | AV403631 | 12 |  | directly mapped |
|  | BM658389 | AY769290 |  | 13 | 13_1 | located on mapped contigs |
|  | BF047034, CA798861 |  | AV400923 | 13 | 13_1 | located on mapped contigs |
| AF122899 |  | AB064522 |  | 13 | 13_2 | located on mapped contigs |
| AY635178 |  | DQ443326 |  | 13 |  | directly mapped |
|  | CA798770, AI187496 |  |  | 13 |  | located on mapped contigs |
|  | BE015609 | AY769301 |  | 13 | 13_12 | directly mapped |
|  | AI172664 | AB195272 |  | 13 | 13_14 | located on mapped contigs |
|  | BE015512 |  | AV402301 | 13 |  | FISH |
|  | AI187470 |  | BY921003 | 13 |  | directly mapped |
|  | BF046807 | AY769315 |  | 13 |  | directly mapped |
|  | BF047031 |  | CK493808 | 13 |  | directly mapped |
|  | BG835807 |  | AV404477 | 13 | 13_18 | located on mapped contigs |
| U12708 |  | AY769316 |  | 14 |  | directly mapped |
| AF062751 |  |  | AV400616 | 14 | 14_8 | located on mapped contigs |
| AF062750 |  |  | CN211399 | 14 | 14_8 | located on mapped contigs |
| AF060795 |  | AB017522 |  | 14 | 14_12 | directly mapped |
|  | BG835804 |  | BP118371 | 14 | 14_12 | located on mapped contigs |
|  | AI187668 | AJ457827 |  | 15 | 15_5 | directly mapped |
|  | CA798699 | AJ490511 |  | 15 | 15_7 | directly mapped |
|  | AI172658 | AY769322 |  | 15 | 15_8 | directly mapped |
|  | BM658433 |  |  | 15 | 15_11 | located on mapped contigs |
|  | CA798757 | AB115083 |  | 15 | 15_11 | directly mapped |
| L78080 |  | AB047924 |  | 15 | 15_a | directly mapped |
|  | EH118273, BG835796 | AY769276 |  | 15 | 15_a | located on mapped contigs |
|  | BM658455, EH118538 | AY769319 |  | 15 | 15_c | FISH |
| AF288088 |  | AF124981 |  | 15 | 15_17 | located on mapped contigs |
|  | BF707436 |  | BP116995 | 15 | 15_17 | located on mapped contigs |
|  | CA483669 |  | CK488562 | 15 |  | directly mapped |
|  | BF046879 |  | BY939231 | 15 | 15_b | located on mapped contigs |
|  | EH118489, CA798814 | AY769272 |  | 15 | 15_b | FISH |
|  | CA798803 | AY769275 |  | 15 | 15_19 | directly mapped |
|  | BF047000 | AB178640 |  | 15 | 15_20 | located on mapped contigs |
|  | BE015595 | AY706958 |  | 15 | 15_24 | located on mapped contigs |
|  | CA798919 | L08106 |  | 15 | 15_24 | located on mapped contigs |
|  | BE015303 | AB016836 |  | 15 | 15_23 | directly mapped |
|  | BG835801, CA798671 | DQ443264 |  | 16 |  | directly mapped |
|  | BF707463 | AY769269 |  | 16 | 16_2 | directly mapped |
| U75307 |  | DQ109670 |  | 16 |  | directly mapped |
|  | CA798787 | DQ311380 |  | 16 |  | directly mapped |
|  | AI187574 |  | BB985260 | 16 | 16_9, 16_10 | directly mapped |
|  | BF047035 | DQ645460 |  | 16 | 16_9, 16_10 | located on mapped contigs |
|  | BF046977 | DQ443365 |  | 16 |  | directly mapped |
| AY672795 |  |  | DC566491 | 16 | 16_11 | located on mapped contigs |
|  | BF046823, EH118203 | AY769273 |  | 16 | 16_16 | directly mapped |
| M79326 |  | AY341912 |  | 16 | 16_18 | located on mapped contigs |
|  | CA798923 | AY769303 |  | 16 | 16_18 | located on mapped contigs |
| AF003253 |  | D49370 |  | 16 | 16_19 | directly mapped |
|  | CA798855 | AY578155 |  | 17 | 17_2 | directly mapped |
|  | CA483682 | AY769291 |  | 17 | 17_3 | directly mapped |
|  | AI187450 | DQ497202 |  | 17 |  | directly mapped |
|  | BF046953 |  | BY938182 | 17 | 17_4 | located on mapped contigs |
|  | BF046858 | AY769299 |  | 17 |  | directly mapped |
| AF323589 |  | AJ251958 |  | 18 | 18_a | directly mapped |
|  | AI142209 |  | CK561983 | 18 |  | directly mapped |
|  | CA798732, CA798719 |  | BY914105 | 18 |  | directly mapped |
| AF103900 |  |  | BP114941 | 18 |  | directly mapped |
|  | BF046853 |  |  | 18 | 18_8 | located on mapped contigs |
| AF487521 |  |  | BP125399 | 19 |  | directly mapped |
| M73798 |  | X94989 |  | 19 | 19_1 | located on mapped contigs |
| M73797 |  | X94988 |  | 19 |  | located on mapped contigs |
| M21797 |  | X94987 |  | 19 | 19_1 | directly mapped |
|  | CA798741 |  | AU005589 | 19 |  | located on mapped contigs |
| AF117599 |  | AJ973405 |  | 19 | 19_8 | directly mapped |
|  | BF046898 | AF509238 |  | 19 | 19_8 | located on mapped contigs |
|  | AI172663, BF047019 | DQ311352 |  | 19 | 19_8 | located on mapped contigs |
|  | BI262622 | DQ311275 |  | 19 | 19_8 | directly mapped |
|  | BF046885 | DQ515926 |  | 19 |  | located on mapped contigs |
| BF046860 | MSC00660 | DQ443396 |  | 19 |  | directly mapped |
| M28820 |  |  |  | 20 | 20_2 | located on mapped contigs |
| AF117595 |  | DQ443423 |  | 20 |  | directly mapped |
| L20096 |  | AY769321 |  | 20 | 20_5 | directly mapped |
|  | BF046847 | DQ443282 |  | 20 |  | directly mapped |
| U43728 |  | AY676608 |  | 20 | 20_9 | located on mapped contigs |
|  | BM658383 | AY769336 |  | 20 | 20_11 | directly mapped |
| AF030547 |  | AB003287 |  | 20 | 20_12 | directly mapped |
|  | BE015426 | AY769333 |  | 21 | 21_2 | located on mapped contigs |
| U17344 |  |  | CK544274 | 21 | 21_3 | directly mapped |
|  | BF046815 | AY706957 | CK489488, CK488133 | 21 | 21_3 | located on mapped contigs |
|  | BE015314 |  | BP178301 | 21 | 21_3 | located on mapped contigs |
| U64795 |  | AY769320 |  | 21 | 21_3 | located on mapped contigs |
|  | BF707456 | DQ311253 |  | 21 |  | directly mapped |
| AF060797 |  |  | CK518341 | 21 |  | located on mapped contigs |
|  | AI142213 | AY769318 |  | 22 |  | FISH |
|  | BF046827 |  | BP183480 | 22 |  | directly mapped |
| M25486 |  | DQ903306 |  | 22 | 22_5 | located on mapped contigs |
| AY007724 |  | D90082 |  | 22 | 22_6 | directly mapped |
|  | CA483733 |  |  | 22 | 22_9 | directly mapped |
|  | CA798955 | AB017550 |  | 22 | 22_10 | directly mapped |
|  | BE015557 |  | CK510334 | 22 |  | directly mapped |
|  | BI262696 |  | CK549734 | 22 |  | located on mapped contigs |
| AF008586 |  | AF529135 |  | 22 |  | located on mapped contigs |
| AF118384 |  | AY864804 |  | 22 | 22_11 | directly mapped |
| AF117587 |  |  | BY943007 | 22 |  | directly mapped |
|  | CA483687 | AB090887 |  | 22 | 22_12 | directly mapped |
|  | CA798823 |  | BY939117 | 23 |  | directly mapped |
|  | BM658405 |  | BY938896 | 23 |  | directly mapped |
|  | CA798873 |  | BY924190 | 23 |  | directly mapped |
|  | CA483685 | DQ311432 |  | 23 | 23_2 | directly mapped |
| L07609 |  | X12978 |  | 23 | 23_4 | located on mapped contigs |
|  | CA798896 | S77508 |  | 23 | 23_4 | located on mapped contigs |
|  | CA798705 | AY426971 |  | 23 | 23_4 | located on mapped contigs |
|  | BE015485 | AY769346 |  | 23 | 23_10 | directly mapped |
|  | CA798935 | AB196701 |  | 23 | 23_11 | directly mapped |
| AF117578 |  | AB196703 |  | 23 | 23_12 | located on mapped contigs |
| S56567 |  | AF098304 |  | 23 | 23_12 | directly mapped |
|  | BF047045 |  | BW999258 | 23 | 23_12 | directly mapped |
|  | CA483714 | DQ443279 |  | 23 | 23_13 | located on mapped contigs |
| DQ094149 |  | BN000691 |  | 23 | 23_14 | located on mapped contigs |
|  | BI262571 |  | BJ985900 | 23 | 23_14 | directly mapped |
| AY232301 |  | AB019538 |  | 23 | 23_15 | directly mapped |
|  | AI142191 | AB201555 |  | 23 | 23_15 | located on mapped contigs |
|  | BF046831, BE015320 | AY461705 |  | 23 |  | directly mapped |
|  | BM658456 | AB196702 |  | 23 | 23_24 | directly mapped |
|  | AI142205 |  | DC543753 | 24 |  | FISH |
|  | BE015464, EH118914 | DQ443196 |  | 24 |  | directly mapped |
| L47123 | MSC01105 | DQ443250 |  | 24 |  | directly mapped |
|  | BF046982 | AB048205 |  | 24 | 24_5 | directly mapped |
|  | BF046892 |  | BJ985752 | 24 |  | directly mapped |
| AY672800 |  | AF513368 |  | 24 | 24_6 | located on mapped contigs |
| AY186577 |  | AY227000 |  | 24 | 24_7 | directly mapped |
|  | BE015326 |  | BY924492 | 24 |  | directly mapped |
| AF327882 |  | AF287267 |  | 25 | 25_1 | directly mapped |
|  | BM658423 | DQ311171 |  | 25 |  | directly mapped |
|  | BF046995 |  | AV398721 | 25 |  | directly mapped |
|  | BF046926 | AY769340 |  | 25 | 25_7 | located on mapped contigs |
|  | BF707451 | AY769345 |  | 25 | 25_7 | directly mapped |
| AY672781 |  |  |  | 25 | 25_8 | directly mapped |
| AY672784 |  | AY061936 |  | 25 | 25_8 | directly mapped |
|  | AY923835, BF046981 |  | BY914040 | 25 |  | directly mapped |
|  | CA798709 |  | BP183449 | 25 | 25_13 | located on mapped contigs |
|  | BF046752 |  |  | 26 | 26_1 | located on mapped contigs |
| AY232302 |  | AB010825 |  | 26 | 26_1 | directly mapped |
|  | BF046915 |  | BP123427 | 26 |  | directly mapped |
| DQ840514 |  | AB255163 |  | 26 |  | directly mapped |
| AY566162 |  | AY566164 |  | P | P_5 | located on mapped contigs |
| AY566166 |  | AY566165 |  | P | P_5 | directly mapped |
|  | CA798822 |  |  | P | P_5 | directly mapped |
|  | BE015381 |  | CK530917 | P |  | directly mapped |
|  | BF707439 | AY706955 |  | P | P_9 | directly mapped |
|  | BM658366 | DQ443278 |  | P |  | located on mapped contigs |
| AJ249388 |  | DQ311199 |  | P |  | directly mapped |
| AY220911 |  | DQ311189 |  | U | U_3 | located on mapped contigs |
|  | BM658362, BG835786 | AB060275 |  | U | U_3 | located on mapped contigs |
|  | BM658407 | AF315317 |  | U | U_7 | directly mapped |
|  | BF046901 | AB195970 |  | U | U_7 | located on mapped contigs |
| U57651 |  | AF309498 |  | U | U_11 | located on mapped contigs |
