# Supplementary material for: Extensive Conserved Synteny of Genes between the Karyotypes of Manduca sexta and Bombyx mori Revealed by BAC-FISH Mapping
Source: PLoS One. 2009 Oct 15;4(10):e7465. doi: 10.1371/journal.pone.0007465 (PMC2759293; doi:10.1371/journal.pone.0007465)
Supplement: Table S3 — Gene anchors in Manduca sexta BACs. Requests for clones or the library should be sent to H.B.Z. Notes: Clones positive for multiple genes are underlined. (0.16 MB DOC) [file pone.0007465.s005.doc]

| **Accession No.** | **Putative function** | **BAC No.** |
| --- | --- | --- |
| AY327249 | ephrin receptor | 15J11, 16B08, 18J11, 19K16, 31A24, 34P04 |
| BE015548 | titin | 42G01 |
| BI262654 | epidermal growth factor receptor | 22D05 |
| AF288089 | betaFTZ-F1 | 40I07 |
| CA798911 | P-glycoprotein | 18A03 |
| BE015311 | NADH dehydrogenase (ubiquinone) | 34M13 |
| CA798718 |  | 19A13 |
| CA483683 | ribosomal protein L17A | 40D12 |
| AY616435 | *Distal-less* | 01G10 |
| AY327250 | ephrin | 49B04 |
| BF707465 | 3-dehydroecdysone 3beta-reductase | 26J07 |
| CA798913 | destabilase 2 | 01E18, 38J11 |
| AI187664 |  | 05G22 |
| BM658435 | ribosomal protein L13A | 36M16 |
| AJ249389 | vacuolar ATPase subunit H | 06J13, 25D01, 25F07, 27I19 |
| BM658406 | ribosomal protein L11 | 06J13, 18B23, 25D01, 25F07, 27I19 |
| AI142211 | glutaredoxin | 18B23, 25D01, 27I19 |
| AI187662 | ADP-ribosylation factor 1 | 02E22 |
| AI187516, AI187517 | mitochondrial porin channel | 15A24 |
| AJ430670 | juvenile hormone diol kinase | 06N05 |
| CA798843 | Mannose-P-dolichol utilization defect 1 protein homolog | 39B08 |
| U44837 | *ultraspiracle* | 19G08, 40N11 |
| BF046761 | Kisir | 09N14, 13M16, 39P16 |
| CA798744, EH118875 | ribosomal protein L3 | 02K20, 09N14, 13M16, 39P16 |
| BF046862 | eukaryotic initiation factor-4a | 09K11, 15M07 |
| BF046854 | prenylated protein tyrosine phosphatase | 40I18 |
| BF046763, CA798832 |  | 39P21 |
| CA798889, EH118892 | eukaryotic translation initiation factor 3 subunit 6 | 30H07, 39I15 |
| CA483695 |  | 18M10 |
| AI172629, AI172630 |  | 46M02, 49K12 |
| BF047058 | ribosomal protein L19 | 01K19 |
| CA798826 | Ribophorin I | 01K19 |
| AI187559 | alpha-4 phosphoprotein | 43G05 |
| AY232304 | antimicrobial protein attacin 2 | 35I17 |
| S77989 | *abd-A* | 49B18 |
| U63300 | *Ubx* | 08L20 |
| U63301 | *Antp* | 30E10 |
| BF047004 | mod(mdg4) | 08F24 |
| AY672792 | hemolymph proteinase 17 | 29A03 |
| CA798909 | ATP synthase subunit b | 29A03 |
| BG835805 | glutathione S-transferase | 29J02 |
| AJ863121 | annexin IX-C. | 13H22, 23C23, 40B18 |
| CA483678, BG835756 | ribosomal protein S27 | 40L08 |
| U02270 | chitinase precursor | 33O19 |
| AF393501 | antennal binding protein 8 | 21N12 |
| AF172845 | death-associated LIM only protein | 40H24 |
| BF046791 | trans-activation-responsive RNA-binding protein | 17L15 |
| AI142161 |  | 17O12 |
| AF032676 | broad-complex | 18G24, 19E10, 27D03, 32C18, 44C17 |
| BF046873 |  | 43M06 |
| BM658430 | vacuolar ATPase subunit a | 24E09, 24I20 |
| AF194819 | heat shock cognate 70 protein | 24E09, 24I20 |
| AI187630 | peptidyl-dipeptidase A | 06L11, 22P16 |
| AY644784 | calcium-activated potassium channel alpha subunit | 09B22, 40D12 |
| AI187592, AI187593 | synaptic vesicle protein SV2 | 08J04, 42I04 |
| BG835772 | ribosomal protein L24 | 09I22 |
| AF053131 | immunolectin-A precursor | 12B03 |
| AI187503 |  | 47F11 |
| U19812 | ecdysone receptor | 17E22, 41F23 |
| BF046895 |  | 42E23 |
| S60738 | E75 B | 18E12, 33K24 |
| AF062749 | nitric oxide synthase | 16P11, 25H05, 32N11, 34P23, 36P24, 49P07 |
| BF046990 | Cu-Zn superoxide dismutase | 32D24 |
| BF046764 | ribosomal protein L14 | 40B02 |
| AI187506, AI187505 |  | 21E22, 21H18 |
| AF177982 | beta-1,3-glucan recognition protein | 42L10 |
| BE015478, BE015477 | diadenosine tetraphosphatase | 22K18 |
| AY172672 | pheromone biosynthesis activating neuropeptide | 36P22 |
| BF046774 | gelsolin | 19E03, 24B10 |
| S71028 | lysozyme | 24P22 |
| BG835758 | ribosomal protein L9 | 28J11 |
| AY368703 | N-acetylglucosaminidase | 29I12 |
| BM658389 | ribosomal protein L21 | 28D18 |
| BE015609 | ribosomal protein L31 | 34K19 |
| AI172664 | heat shock protein 27 | 27O16 |
| BE015512 | COP9 complex subunit 3 | 35H08 |
| BG835807 |  | 36C12 |
| U12708 | ribosomal protein S3 | 25L03 |
| AF062751 | guanylyl cyclase beta-1 subunit | 05H01, 22M15 |
| AF062750 | guanylyl cyclase alpha-1 subunit | 37I09 |
| BG835804 | msps | 06J08 |
| AI187668 | ribosomal protein P0 | 02I22 |
| AI172658 | ribosomal protein S8 | 02B17, 02P16 |
| BM658455, EH118538 | ribosomal protein S5 | 20M01 |
| AF288088 | hormone receptor 4 | 09P16 |
| BF707436 | translation initiation factor 3 subunit 2 | 19P12 |
| CA798803 | ribosomal protein L7A | 30J18, 33N21 |
| BE015595 | ribosomal protein S23 | 09A17, 14B04 |
| CA798919 | glycyl-tRNA synthetase | 14B04 |
| BE015303 | heat shock 70 kD protein cognate | 30P01, 33N13 |
| BG835801, CA798671 | phosphatidylethanolamine-binding protein | 40L06 |
| AI187574 | methyltransferase | 24J16 |
| BF047035 | eukaryotic translation initiation factor 3, subunit 8 | 45I01, 49P16 |
| AY672795 | hemolymph proteinase 19 | 23K13 |
| M79326 | apolipophorin-III | 16D15 |
| AF003253 | pro-phenol oxidase subunit 1 | 02A12, 05D20 |
| AI187450 | translation initiation factor 3 beta | 43N08 |
| BF046953 |  | 06P06 |
| BF046858 | ribosomal protein L29 | 26P10 |
| AF323589 | sensory neuron membrane protein 1 | 47E07 |
| AI142209 | inositol 1,3,4-trisphosphate 5/6-kinase | 10J18 |
| CA798732, CA798719 |  | 44A16 |
| AF103900 | fasciclin II transmembrane isoform | 05E16, 11N12, 14E02, 18K01, 31E02, 36G11, 37M06, 40I11, 49N09 |
| AF487521 | cuticle protein 36a | 23K02, 31A15 |
| M73798 | general odorant-binding protein 2 | 36C03 |
| M21797 | pheromone-binding protein | 36C03 |
| AF117599 | sensory appendage protein 4 | 21O24 |
| AI172663, BF047019 | APC-binding protein EB1 | 35M12 |
| BF046860 | elongation factor 2 | 15P18 |
| M28820 | microvitellogenin | 09H14 |
| AF117595 | FK506-binding protein | 45N18 |
| L20096 | ribosomal protein S7 | 28O14 |
| BF046847 | cytochrome c oxidase subunit Vb | 06J09, 06P21, 36M21 |
| BM658383 | ribosomal protein S20 | 10O14, 37P12 |
| BE015426 | ribosomal protein S17 | 40G12 |
| U17344 | bumetanide sensitive NaK_2_Cl cotransporter | 23A13 |
| BF046815 | ribosomal protein S15 | 15M04, 23A13, 49B03 |
| BE015314 |  | 23A13, 23F19 |
| U64795 | ribosomal protein S6 | 23G23, 47C12 |
| BF707456 |  | 06P07 |
| AF060797 | beta-ureidopropionase | 06P07 |
| AI142213 | ribosomal protein S4 | 40N16 |
| BF046827 | cuticular protein | 39L20 |
| M25486 | larval cuticular protein | 37N24 |
| AY007724 | prothoracicotrophic hormone | 37I17 |
| AF008586 | dihydrolipoamide dehydrogenase (E3) | 06A23, 06I24 |
| AF118384 | N-ethylmaleimide sensitive fusion protein and Hitcher protein | 41A13 |
| CA483687 | calreticulin | 25I24 |
| CA798823 | small ribonuclear protein G7b | 35M17 |
| BM658405 |  | 39L03 |
| L07609 | methionine-rich storage protein 1 | 21A19 |
| BE015485 | ribosomal protein S30 | 29A18 |
| CA798935 | hemolymph juvenile hormone-binding protein precursor | 19L19 |
| AF117578 | juvenile hormone binding protein | 25A10 |
| BF047045 |  | 25A10, 26A14 |
| AY232301 | antimicrobial peptide moricin | 21D05, 29N07 |
| BM658456 |  | 12B09 |
| AI142205 |  | 33D22 |
| BE015464, EH118914 | ferritin subunit 1 | 05A16, 31E23 |
| AY672800 | serine proteinase-like protein 4 | 31P12, 32D17 |
| AY186577 | ADP/ATP translocase | 35B16 |
| AF327882 | juvenile hormone esterase | 22D07 |
| BM658423 | electron transfer flavoprotein beta-subunit | 28C10 |
| AY923835, BF046981 | sodium pump alpha subunit | 37H22 |
| CA798709 | mitogen inducible gene mig-2 | 49G18 |
| BF046752 |  | 24N15 |
| AY232302 | antimicrobial peptide cecropin 6 | 15J08, 49G02 |
| BF046915 | NAD(+)-isocitrate dehydrogenase subunit 1 | 45G05 |
| DQ840514 | octopamine receptor | 28A19, 28M21 |
| AY566162 | serpin-4A | 05E05 |
| CA798822 |  | 26P13, 42F24 |
| BE015381 |  | 10I15 |
| BF707439 | ribosomal protein S11 | 17D24 |
| AJ249388 | vacuolar ATPase subunit C | 22H07 |
| AY220911 | heat shock protein 70 | 17E20 |
| BM658362, BG835786 | 90-kDa heat shock protein HSP83 | 17E20, 42O03 |
| BM658407 | heat shock protein hsp20.8A | 08O10 |
| BF046901 | alpha-crystallin cognate protein 25 | 08H21 |
| U57651 | apolipophorin precursor protein | 12L18 |
