# Supplementary material for: Extensive Conserved Synteny of Genes between the Karyotypes of Manduca sexta and Bombyx mori Revealed by BAC-FISH Mapping
Source: PLoS One. 2009 Oct 15;4(10):e7465. doi: 10.1371/journal.pone.0007465 (PMC2759293; doi:10.1371/journal.pone.0007465)
Supplement: Table S4 — STS primers used for PCR screening of the Manduca sexta BAC library. (0.16 MB DOC) [file pone.0007465.s006.doc]

| **Accession No.** | **Forward primer sequence** | **Reverse primer sequence** | **Product Size** |
| --- | --- | --- | --- |
| AY327249 | ATTCCCAGCAACACCTACA | AGCAAAAGTCACAGTCAAG | 397 |
| BE015548 | TTCTAAGGAGCCGATGT | CTCTAAGCCAGTAAACG | 119 |
| BI262654 | GACTTCCGTCCTATTCTAC | AGCGATTCTGAGCCTTCCA | ca. 170 |
| AF288089 | GAAACGACGCTCCATAAC | GGCACCACCATCACCAGT | ca. 800 |
| CA798911 | CGTGCTGAAAGGGATAGA | CTGAGCCGAGGTAAGGTG | ca. 1800 |
| BE015311 | GCAAACAAGCAGAACTAAAA | AATCATTGACATAAACCA | 284 |
| CA798718 | GGTGAGGATAAGTTTGG | GTGGCAGATCAGTTTTGT | ca. 1200 |
| CA483683 | CGAAGTTCCGCATCTCAC | GGCAATCACATACAGGTT | ca. 200 |
| AY616435 | TTCCACCCCCACTAATGT | ACCAGGACTGCCAAAATG | 112 |
| AY327250 | CCGTGTCGCAGTGTTAGTTT | TTTCCGTGTCTTCCTCGTAG | 397 |
| BF707465 | CCTTTTCGTTTGACTTTGAC | CCCTGTTTTATCTTCTGGTT | 154 |
| CA798913 | GCTCCCCTTCAACTACG | GGCACATCCTTCAACTCT | 106 |
| AI187664 | GATATTAACCCGCTTTG | ATCTTGACGAACTGAGG | 124 |
| BM658435 | AAGTTGTTGTTGTTCGTTGC | GATTTTGGATGGTGCTCTGA | ca. 380 |
| AJ249389 | GCCACGACGACCCCAATG | GCAACAATAACAACAAAC | ca. 770 |
| BM658406 | GCAGCCTGTATTCTCTAAG | CAACTCGTATTCTCTCACT | ca. 1000 |
| AI142211 | CGACGCTATTCAAGACAA | CAACGCAGTTACCATTTA | ca. 1300 |
| AI187662 | TGGATTCAATGTGGAGA | ACCCTGTGTATTCTGGA | 124 |
| AI187516, AI187517 | GCGAATCTGGTGTGGAAT | GTCTGTGGTGCGAAGGTC | ca. 840 |
| AJ430670 | AGAAGAAGCTCCTCCAC | GCAGATCAAACAAAAAT | 548 |
| CA798843 | TGAAGGAACATTTTTAGCA | GCAACACCAGAGCACCATT | ca. 650 |
| U44837 | GCCTCCTACATACACGAC | AGACCCAACACGCCTTTA | 271 |
| BF046761 | TGAAAAGTCTGAAGTGAACC | AGAGGGATGGGGAGTGTAT | 115 |
| CA798744, EH118875 | GATTGATTGTATTGGTGT | AGTCCTGTGATGGTAGCCC | ca. 390 |
| BF046862 | GGAGCCCTGGATACTAAC | ATAAGAGCCTGACATTCG | 252 |
| BF046854 | ATGGGCACAAGAACAACT | CGGTGAAATGATACAAAA | 203 |
| BF046763, CA798832 | CTGCCCCAAAAACTCAAT | GGTCATAGAATCGGAAGC | 150 |
| CA798889, EH118892 | TAAGTTGGCGAGCGAAAT | GTTGAAGAAGACGAAGAG | 169 |
| CA483695 | AGATTGTCCTGGTGGTGAG | AGTTCGTCCGCTAAAGTGA | ca.350 |
| AI172629, AI172630 | GCAAAAAGTGTGATTAGGC | GAAAGGTTCGGGGTCTGA | 178 |
| BF047058 | CTGCTTCTGATGTTTACTAT | TCTTGGATGTGGTTTTCTAC | 409 |
| CA798826 | GCAAACACCAACTCCAG | CTTCTCAGCCTTCTTCC | ca. 1300 |
| AI187559 | AAGCATAAAAGCAGCAATA | TGAGAGTTCCCGAGCAGTG | 147 |
| AY232304 | TCGGTGGGGCTGATTTTA | TGTTGTTGTGGAAGAGGT | ca. 470 |
| S77989 | GCGGCAAACCTACACAA | CATGCGCCGATTCTGGA | 151 |
| U63300 | ACGAGGAAGACAAACG | CAACAACCGAACAACA | 406 |
| U63301 | TCGAGCTGGAGAAGGAG | GGACCGCGTGAACTAAG | 233 |
| BF047004 | GAATCCAACTCAACATCC | CGTCATCCCACATACTTT | 515 |
| AY672792 | GACTTAGTGCGAGATGACGA | TAAAACGAGGATGCCGATGT | 117 |
| CA798909 | TGCGCGATGAGAAGAACT | TGAGGAGGATGGAGAGAC | ca. 620 |
| BG835805 | GCTGGCAGGGGACGAAGT | CAGGCTGTGAGTTTTGGA | ca. 1300 |
| AJ863121 | CCAACCGTGATGAGAACCAG | TCGTGTGATGAGAATAGAGT | 128 |
| CA483678, BG835756 | GCCACATCCCAACTCGTA | CAAACAACAACCCTCTGC | 93 |
| U02270 | AAGAAATGGGACGAGCAAGG | GTATGAGGAGGCGGCACTGT | 239 |
| AF393501 | ACTCAAGGCTGGCGACTAT | GCTCCGACTTCATCATCA | ca. 680 |
| AF172845 | GACAACAAACCACTCTGC | TAGGCTTAATCACTTCAA | 662 |
| BF046791 | CTCGCAGACGCTATCAAA | ACTCGGTAACGGAAGGTG | ca. 1100 |
| AI142161 | GCGTATAAATCGGAACAT | TTGCCAGAGTGAAAGTGC | 126 |
| AF032676 | AGCGGATGCTGAAGACC | CTGGATGCTGAGTGAGG | 131 |
| BF046873 | GAAATCCCACAAAATACT | GTTCTTACAGCCAGCATA | ca. 340 |
| BM658430 | AACTACAACGCCAGCACT | ACATACGCCGAACAACAT | ca. 700 |
| AF194819 | TGTTTGCTGAGTTTGTTC | GCCCACGCACGAGTATGT | ca. 700 |
| AI187630 | GCCGCTGGGAGGGAGTA | TCTTGAGGTCTTCGTCTTC | ca. 500 |
| AY644784 | GAGAGTGGAAGATGTTGC | CGTGGGGTCGTCGTTGGA | ca. 380 |
| AI187592, AI187593 | AGCATAGATTCCCCAACA | CAGAGAGGCAACCACAGC | 175 |
| BG835772 | AGAAATCCTCGTAAAGTAA | GGCACCAACAATAGCAC | ca. 520 |
| AF053131 | TACCAGACGAAGCAATACCG | TCCGCCACCCACACATAGTT | 218 |
| AI187503 | GCATAGGTTTTTCTGTAG | ACCAAAAAGTGATAACAAAA | 214 |
| U19812 | TTCTTCAGGCGGAGTGTG | CTGGCAGTTTGTCTTTTT | 214 |
| BF046895 | TAACGAAAACCCCAGATAAA | TAAAACGCCATAGTGTAAGC | 162 |
| S60738 | TCTTTGACGCTCTATTTG | CCTCCTCGCTCCACATTT | 453 |
| AF062749 | TACAAAGCCTCAAACCAG | CATCCGAAGAACAACCAC | ca. 440 |
| BF046990 | ATCCCACTGACGAGAACC | ATGACACAACAAGCAACC | 229 |
| BF046764 | GCCTCAACCAACTTCATC | GCCTTCACCTTTAGACTC | 250 |
| AI187506, AI187505 | CGCATCCTATCTCTCATCT | TCTCCAATCATCAAAGTTC | 225 |
| AF177982 | TTCCACGGCAAACTCAAT | TACCCCAACCCATCCTTA | 170 |
| BE015478, BE015477 | CGTCAGGCACCATTCAACA | GGCTTCTCCTTCACCATCT | 300 |
| AY172672 | GTCTCGGCAGGGAACTTA | AAAACACTGGCGTAGGTA | 496 |
| BF046774 | GCAGACAAAAGGAACAAC | CACTCTCGTAGCCCAAAG | 160 |
| S71028 | TGCCGACGACTTCTCAATC | CTCACAATCCCTCATCAAA | 151 |
| BG835758 | AGCAAATAGTGGCAAACC | ACCTTCAACTGGCGAGAG | 160 |
| AY368703 | CCCTGGAAATGGACGAGA | TGACGAATGCCGAAGAAC | 100 |
| BM658389 | GCTCTCGGCGTAATCGTG | CTCTTCAGGTGGACAACT | 179 |
| BE015609 | ACCCAAAGGCGAGAGGA | TTGTTTAGGCGAGTGTC | 192 |
| AI172664 | CCATCAAGTCATCGCTGTC | ACTTTTAAGAATCGCTGTG | 346 |
| BE015512 | TTGGTGTCACTCATTTTA | GTGTATTTTGGCATTGGT | 310 |
| BG835807 | AAATCATATCCGCCACCAC | GAACTCTCCCATCCGACTG | 103 |
| U12708 | TGGCTGAGGATGGCTACT | CACGGGTGGCAACTTTCT | 198 |
| AF062751 | GAGTGTTTCCATTCCATTT | GACGCACAGTATCCAAGAC | 132 |
| AF062750 | CAGAAGAAGAAGATGGAGAG | GATGGAGCAGTCAGGAGTTG | 79 |
| BG835804 | GCCCTAAGTCGCTCTAAG | GGAAACTCGCCTCTATGC | ca. 430 |
| AI187668 | AGGGAGGACAAGGCTACT | CCACGGGTGAAGACAAAG | ca. 800 |
| AI172658 | GCAAGAAGAGGAAGTATGA | CTGAACCCCACGAGAAAT | ca. 280 |
| BM658455, EH118538 | AACAGCCATTATCAACTC | CCAACTCATCTTTCTTCT | 251 |
| AF288088 | ATACGCCAACGCCTCCAA | GTCTCCAGCCCATAACAG | 137 |
| BF707436 | CGTCGTGTGGTGCTTAGA | TGGCTTTGTCAGTAGTAT | ca. 600 |
| CA798803 | GAAGAAGGTCGGGAAGAA | TGAACTGGTTGATTGGTG | ca. 850 |
| BE015595 | GAAGAGAACGACGAAATG | CAAGGTTAGGAGGGACAC | ca. 420 |
| CA798919 | TACCCTACGCCATCACAGTT | GCTCCTTTTACAGTCTCTTG | 205 |
| BE015303 | GTATCATCGCTGGTCTCA | GCCCTTCTTCTTCTTGTA | 263 |
| BG835801, CA798671 | CGTCCCAATACTTTACAA | CCAGCATTCCGCCGTCTAC | 234 |
| AI187574 | GTCGCAGTAGAAAGAGAAA | GCCCCATCAAAACAACCAG | 89 |
| BF047035 | ACTGAGGATAATGAGGAAT | CAATGCGTGATGGTAGATA | 414 |
| AY672795 | GCGGTGGCTGTTAGATTC | TCGTTGACTGAGGCTTGA | 199 |
| M79326 | GCGTTTGAAGAGATGGAGAA | TGGGTGTTTTTGGAGTTGAC | 89 |
| AF003253 | CTACACCCACGACCCTGAT | CGACGAACCGATGCCAACG | 507 |
| AI187450 | GACCAAAAACCCAAGGAATC | CCAAGAAAATGTAGCCAGTG | 190 |
| BF046953 | AACTAAAACAACAACGAG | TTCTTCTTCAACAACAAT | 136 |
| BF046858 | CCACACAAATCATAACCA | TCCATCCCAAGAGTAGAC | 246 |
| AF323589 | TAGGGAAGGCGATAAATGG | TGGGCTCAATAATAAGACT | 177 |
| AI142209 | GCTGGACTTGGATAGGC | CATTCGGATGATTTGACA | ca. 230 |
| CA798732, CA798719 | GTAGCACCCCAGCAACAA | GAATAAGAGTAGCCATCG | 296 |
| AF103900 | ACATACGACTTTAGGTTC | CGGCACTTTCCACTTCAG | ca. 300 |
| AF487521 | AACAATGAGGTGACAGC | CGGGGTGGGGAGGTGAG | 204 |
| M73798 | TGGAGGTGATGGATGAGTT | ATGTCGTCGTATTGCTTCT | ca. 760 |
| M21797 | CGAAGCAATGGCGAAGCAAC | CTCCGTGTAGTATCTCAAAA | 513 |
| AF117599 | GAAAGGCTATGTGGATTG | AGGTTGTTAGGGTCGTAT | 210 |
| AI172663, BF047019 | ACTGGTGAAGGGTCGTTT | AGATTTACTGGGGGAGAT | ca. 220 |
| BF046860 | GCTTCACCGCCGACTTGC | TCCTTTCCTCTTTCTTGT | ca. 500 |
| M28820 | CAGCGAAACACTATGGAA | CTCAGAAAGGGGAACAAA | 249 |
| AF117595 | TTGGCAAGGGAGAGGTGA | AGAATGTTTGTGGGTGAG | ca. 420 |
| L20096 | TGCCGATGCCCAAACTGAA | GTCTCCAATGAACACCACA | 101 |
| BF046847 | TGGTATTGAAAGACAGGA | AAGCATGAGGGCACGAG | 120 |
| BM658383 | GCCAATCCACCGCATCAG | GACCTCAACCTCTACACCA | ca. 460 |
| BE015426 | CACTTGATGATGCGTCTC | CCACCGTAACCACCTTGT | 224 |
| U17344 | TATCATTACTCGCCCTTTA | GTCTTTTCAACGCTCTTTT | 185 |
| BF046815 | AGCCGATGGCGTTAGTCA | AATGTCTTTCCGTTGTAG | 151 |
| BE015314 | ATAGAACACAGCAAGCAGT | GCAGATGTCCGCCTCAGC | ca. 420 |
| U64795 | GCTCCGTATCTTCTATGA | GACTTGCGTTTCCTTTCG | ca. 470 |
| BF707456 | AGGGACCAAATAGAAATG | CTTGTAAACACGCTCACC | ca. 640 |
| AF060797 | GGACAACGAAACGCAGAG | TTGGATAACGCCTACCTT | ca. 430 |
| AI142213 | TAAACGCACCGAAAGCAT | ATCACCAAAGGCAGACAC | 103 |
| BF046827 | CGCTTACGTCTATGGAT | ACTTTGGGAATGTTGTCT | ca.370 |
| M25486 | CATTGCTTTTCTGTGATTG | TCTTCGGGGGCTCTGGTTC | 471 |
| AY007724 | ATTATGTTCCTGTATCTTCA | TGTTGCCTCTTTTCCGTGTTC | 317 |
| AF008586 | CCCATCAAAAGCCCTCCTC | GAACAGCATAGCAATACCC | 166 |
| AF118384 | GAATCCGCTAAATGCTTG | CTGCCCCGAGGTGGTTGT | ca. 410 |
| CA483687 | AGTCAGGCACCATCTTTG | TCGCTCTTCATCTTCTTC | 112 |
| CA798823 | GAGGGTCTGTGGTTCGTT | GCTGTTTATAGGTTAGGA | 119 |
| BM658405 | AAGTATGAGTTTGATTTGGA | CGCTGTGGTGGGGTATGTTA | ca. 800 |
| L07609 | CCAACTACTACGGCATCA | GCGAAGCATCCTCTCAAC | 446 |
| BE015485 | TGCTAACATTGAAGGTGA | GGGTATTGTGAGGTCCAG | 109 |
| CA798935 | GAATGTGCTCTGAAATC | TTGAAGCCACTCTCGTTG | 134 |
| AF117578 | TCCTCACCTTCTTGTTTAC | ATTTCTCCATTGTCCTACG | 359 |
| BF047045 | ACTGCCCGTGTTCCTTGAT | TCCTTTTAGTTTCCCTTCTG | 127 |
| AY232301 | GAAAAGGTCTACGAGCAATAAA | AAAGATGAATAATAAACGATG | 424 |
| BM658456 | TCAGAGCAAAGGTAAGCA | CGGATGTTGATGTGGTAT | 126 |
| AI142205 | ATGATGTGAGCGAAAATAAG | TCTCCGAACAAAAATAACCT | 192 |
| BE015464, EH118914 | CGTCACCTCGCTCATACAA | CGTCGCTCTCCATCTTCAG | ca. 320 |
| AY672800 | GAGCCCTATCCCCACCAG | TCTTTGCCGAACTTGTCC | ca. 700 |
| AY186577 | GCAAGGTATCATCATCTA | ATAGTCGCCCAGCAGTG | ca. 220 |
| AF327882 | CATCTGCTTTCTTTACCAC | CGTTACTGCGGGCTCTCTT | 555 |
| BM658423 | GGAGGAGGCAGTCAGGTT | ACAGTTTGGCGAGGATTT | ca. 400 |
| AY923835, BF046981 | ATCGTGACTGGCGTAGAGG | CACAGGATAAAGGCAAGGA | 116 |
| CA798709 | CTTATGTCCAGCCACCAGT | GCAAAGTAGTTCACGAGTT | 117 |
| BF046752 | TGGATGGGGCTGACTTGGTG | GGCTTGGCTGCTTTTACTGA | ca. 1050 |
| AY232302 | ATTCTCCCGTGTTTTATT | CTTCTTTTGATTGTCCTTT | ca. 750 |
| BF046915 | GCCACGCCTGACTTCTCC | GCTCTCTGATGATGATGC | 154 |
| DQ840514 | CGTATCCTATCCTTCATCA | TAGTCCTTGGTTTATCATC | 203 |
