# Supplementary material for: Extensive Conserved Synteny of Genes between the Karyotypes of Manduca sexta and Bombyx mori Revealed by BAC-FISH Mapping
Source: PLoS One. 2009 Oct 15;4(10):e7465. doi: 10.1371/journal.pone.0007465 (PMC2759293; doi:10.1371/journal.pone.0007465)
Supplement: Table S5 — Summary of BAC-FISH analysis. * G, O, R, C5: BAC probes labeled with Green-dUTP, Orange-dUTP, Red-dUTP and Cy5-dUTP and pseudocolored for green, yellow, red and cyan if not specifically mentioned; - represents BAC not used in Fig. 1 but in Fig. 2. ** According to Kaikobase (http://kaikoblast.dna.affrc.go.jp/) a The upstream region was misintegrated to chr. 13 in Kaikobase. b The upstream region was mistakenly integrated to chr. 3 in Kaikobase. (0.25 MB DOC) [file pone.0007465.s007.doc]

| **Accession No.** | ***M. sexta*** | | | | ***B. mori*** | | |
| --- | --- | --- | --- | --- | --- | --- | --- |
|  | **BACs used** | **location** | **FISH probes*** | | **Chr.** | | **location in Kaikobase^**^** |
|  |  |  | **for karyotype (Fig. 1)** | **for gene ordering  (Fig. 2)** |  |  |  |
| AY327249 | 15J11 | Z_1 | O | G | 1 | 498545-501746 | |
| BE015548 | 42G01 | Z_2 | − | C5 | 1 | 1606975-1604490 | |
| BI262654 | 22D05 | Z_3 | O | O | 1 | 5821662-5821358 | |
| BE015311 | 34M13 | Z_4 | − | R | 1 | 19208582-19208743 | |
| CA798911 | 18A03 | Z_5 | G | G | 1 | 12184767-12185495 | |
| BF707465 | 26J07 | 2_1 | O | C5 reprobed / purple | 2 | 8025981-8042831 | |
| AY327250 | 49B04 | 2_2 | C5 | G | 2 | 6327255-6326542 | |
| AY616435 | 01G10 | 2_3 | − | data not shown | 2 | 5891342-5771690 | |
| DQ840514 | 28A19 | 2_4 | − | R | 26 | 7440062-7471334 | |
| AY232302 | 49G02 | 2_5 | O | O | 26 | 11369548-11369459 | |
| BF046915 | 45G05 | 2_6 | − | C5 | 26 | not mapped in Kaikobase | |
| CA798913 | 38J11 | 3_1 | − | O | 3 | 3764986-3760993 | |
| AI187664 | 05G22 | 3_2 | − | G | 3 | 7445383-7448706 | |
| BM658435 | 36M16 | 3_3 | − | R | 3 | 8883278-8885694 | |
| AJ249389 | 18B23 | 3_4 | O | C5 | 3 | 9613444-9620082 | |
| BM658406 |  |  |  |  | 3 | 9673259-9676551 | |
| AI142211 |  |  |  |  | 3 | 9832811-9835577 | |
| AI187516, AI187517 | 15A24 | 3_5 | R | O reprobed | 3 | 12376563-12372690 | |
| AJ430670 | 06N05 | 3_6 | O | G reprobed | 3 | 17355147-17355756 | |
| CA798843 | 39B08 | 4_1 | G | O | 4 | 4715276-4716033 | |
| U44837 | 19G08, 40N11 | 4_2 | R | C5 | 4 | 6417495-6451584 | |
| BF046761 | 13M16 | 4_3 | − | G | 4 | 10799940-10800541 | |
| CA798744, EH118875 |  |  |  |  | 4 | 10814055-10814738 | |
| BF046862 | 09K11 | 4_4 | G | R | 4 | 13065794-13062463 | |
| BF046854 | 40I18 | 5_1 | G | O | 5 | 16890078-16892815 | |
| BF046763, CA798832 | 39P21 | 5_2 | C5 | G | 5 | 14561847-14562761 | |
| AI172629, AI172630 | 49K12 | 5_3 | − | C5 | 5 | 6112467-6113309 | |
| AI187559 | 43G05 | 5_4 | G | R | 5 | 1962024-1960352 | |
| AY232304 | 35I17 | 6_1 | G | G | 6 | 18597848-18596848 | |
| U63300 | 08L20 | 6_2 | O | C5 | 6 | 6814659-6814417 | |
| BF047004 | 08F24 | 6_3 | G | O | 6 | 4597067-4596419 | |
| AY672792 | 29A03 | 7_1 | − | G | 7 | 14863562-14837408 | |
| CA798909 |  |  |  |  | 7 | 14785446-14782397 | |
| BG835805 | 29J02 | 7_2 | C5 | C5 | 7 | 11298881-11298023^a^ | |
| U02270 | 33O19 | 7_3 | G | O | 7 | not mapped in Kaikobase | |
| AF172845 | 40H24 | 8_1 | − | G | 8 | 858993-849974 | |
| BF046791 | 17L15 | 8_2 | G | C5 | 8 | 7490308-7488058 | |
| AI142161 | 17O12 | 8_3 | G | O | 8 | 13203335-13204152 | |
| AF032676 | 18G24 | 8_4 | − | R | 8 | 18077270-18088253 | |
| BF046873 | 43M06 | 8_5 | G | O reprobed / purple | 8 | 18362091-18363370 | |
| BM658430 | 24E09 | 9_1 | − | C5 | 9 | 17679685-17681536 | |
| AF194819 |  |  |  |  | 9 | 17635253-17631867 | |
| AI187630 | 06L11 | 9_2 | O | O | 9 | 17253191-17254105 | |
| AY644784 | 09B22 | 9_3 | C5 | R | 9 | 15735668-15661609 | |
| AI187592, AI187593 | 42I04 | 9_4 | C5 | G | 9 | 13603267-13600271 | |
| BG835772 | 09I22 | 9_5 | − | C5 reprobed / purple | 9 | 7899229-7900076 | |
| AI187503 | 47F11 | 10_1 | − | O | 10 | 5639900-5639271 | |
| BF046895 | 42E23 | 10_2 | − | G reprobed | 10 | 6137889-6136798 | |
| S60738 | 18E12 | 10_3 | C5 | C5 | 10 | 8493033-8476933 | |
| AF062749 | 16P11 | 10_4 | R | G | 10 | 14979300-14942013 | |
| BF046990 | 32D24 | 10_5 | C5 | R | 10 | 17021383-17019685 | |
| BF046764 | 40B02 | 11_1 | − | R | 11 | 14984367-14984789 | |
| AF177982 | 42L10 | 11_2 | R | G | 11 | 8143711-8157423 | |
| BE015478, BE015477 | 22K18 | 11_3 | R | C5 | 11 | 6321704-6320023 | |
| S71028 | 24P22 | 12_1 | − | G | 12 | 3312581-3308427 | |
| BG835758 | 28J11 | 12_2 | R | O | 12 | 7920434-7922948 | |
| AY368703 | 29I12 | 12_3 | C5 | C5 | 12 | 17880646-17841282 | |
| BM658389 | 28D18 | 13_1 | C5 | R | 13 | 16216987-16214505 | |
| BE015609 | 34K19 | 13_2 | − | G | 13 | 9474668-9476192 | |
| AI172664 | 27O16 | 13_3 | R | O | 13 | 8834720-8834827 | |
| BG835807 | 36C12 | 13_4 | C5 | C5 | 13 | 3599225-3592816 | |
| U12708 | 25L03 | 14_1 | O | O | 14 | 14116030-14112558 | |
| AF062750 | 05H01 | 14_2 | G | data not shown | 14 | 8767514-8824733 | |
| AF062751 | 37I09 | 14_3 | − | G | 14 | 8970470-8981169 | |
| BG835804 | 06J08 | 14_4 | − | R | 14 | not mapped in Kaikobase | |
| CA798823 | 35M17 | 15_1 | − | data not shown | 23 | 21738052-21737768 | |
| BM658405 | 39L03 | 15_2 | C5 | C5 reprobed / purple | 23 | 22463301-22460714 | |
| AI187668 | 02I22 | 15_3 | C5 | O | 15 | 16149748-16148852 | |
| AI172658 | 02B17 | 15_4 | − | data not shown | 15 | 13998337-14000447 | |
| BM658383 | 10O14 | 15_6 | − | R reprobed / light blue | 20 | 13290369-13287099 | |
| BF707436 | 19P12 | 15_7 | − | R | 15 | 5019155-5017684 | |
| CA798803 | 30J18 | 15_8 | C5 | O reprobed | 15 | 2912712-2916685 | |
| BE015595 | 09A17 | 15_9 | − | G | 15 | 1859732-1862279 | |
| BE015303 | 30P01 | 15_10 | − | C5 | 15 | 1591227-1590721 | |
| BG835801, CA798671 | 40L06 | 16_1 | O | R | 16 | 12491599-12490079 | |
| BF047035 | 49P16 | 16_2 | C5 | G | 16 | 5560399-5567140 | |
| AY672795 | 23K13 | 16_3 | − | data not shown | 16 | 2939372-2926270 | |
| M79326 | 16D15 | 16_4 | − | O | 16 | 9819-8677^b^ | |
| AF003253 | 02A12 | 16_5 | O | C5 | 16 | 328799-338813 | |
| AI187450 | 43N08 | 17_1 | − | O | 17 | 13657304-13657552 | |
| BF046953 | 06P06 | 17_2 | O | C5 | 17 | 12253603-12249409 | |
| BF046858 | 26P10 | 17_3 | − | G | 17 | 4315397-4311195 | |
| AF323589 | 47E07 | 18_1 | G | O | 18 | not mapped in Kaikobase | |
| CA798732, CA798719 | 44A16 | 18_2 | C5 | G | 18 | 8684532-8683693 | |
| AF103900 | 11N12 | 18_3 | − | C5 | 18 | 10140424-10136441 | |
| AF487521 | 23K02 | 19_1 | − | O | 19 | 487590-487895 | |
| AF117599 | 21O24 | 19_2 | − | R | 19 | 7396635-7397012 | |
| AI172663, BF047019 | 35M12 | 19_3 | O | C5 | 19 | 7594209-7591907 | |
| BF046860 | 15P18 | 19_4 | O | G | 19 | 12804162-12802874 | |
| M28820 | 09H14 | 20_1 | − | R | 20 | 4065050-4065852 | |
| L20096 | 28O14 | 20_2 | R | G | 20 | 5819521-5815810 | |
| AF117595 | 45N18 | 20_3 | G | C5 | 20 | 4287541-4288337 | |
| BF046847 | 06P21 | 20_4 | − | O | 20 | 9912801-9916057 | |
| BE015426 | 40G12 | 21_1 | C5 | O | 21 | 6330515-6328769 | |
| BF046815 | 15M04 | 21_2 | C5 | G | 21 | 6721368-6718236 | |
| BF707456 | 06P07 | 21_3 | − | R | 21 | 13345714-13347886 | |
| AF060797 |  |  |  |  | 21 | 13386897-13387448 | |
| AY672800 | 31P12 | 22_1 | G | O | 24 | 14446795-14461996 | |
| BE015464, EH118914 | 05A16 | 22_2 | O | C5 reprobed | 24 | 13276301-13257941 | |
| BF046827 | 39L20 | 22_3 | − | G | 22 | 18612350-18614193 | |
| AY007724 | 37I17 | 22_4 | − | O reprobed | 22 | 10389791-10392060 | |
| M25486 | 37N24 | 22_5 | O | C5 | 22 | 9782714-9782929 | |
| AF008586 | 06A23 | 22_6 | − | data not shown | 22 | 7835792-7844477 | |
| AF118384 | 41A13 | 22_7 | − | G reprobed | 22 | 6644577-6639830 | |
| CA483687 | 25I24 | 22_8 | − | R | 22 | 4686951-4685552 | |
| L07609 | 21A19 | 23_1 | − | C5 | 23 | 16910042-16906460 | |
| CA798935 | 19L19 | 23_2 | R | O | 23 | 11309611-11305305 | |
| BF047045 | 26A14 | 23_3 | − | G | 23 | 11124630-11124025 | |
| AY232301 | 29N07 | 23_4 | O | O | 23 | 9241196-9240122 | |
| BM658456 | 12B09 | 23_5 | R | R | 23 | 348015-353377 | |
| AI142205 | 33D22 | 24 | C5 | C5 | 24 | 2084060-2089078 | |
| AF327882 | 22D07 | 25_1 | − | O | 25 | 419918-401929 | |
| BM658423 | 28C10 | 25_2 | O | R | 25 | 4372602-4374319 | |
| AY923835, BF046981 | 37H22 | 25_3 | G | C5 | 25 | 10294838-10295538 | |
| CA798709 | 49G18 | 25_4 | − | G | 25 | 12213552-12210131 | |
| BF046774 | 19E03 | 26_1 | G | G | 11 | 1328189-1332201 | |
| AY172672 | 36P22 | 26_2 | O | O | 11 | 2425019-2421800 | |
| AY220911 | 17E20 | 27_1 | − | O | 27 | 12565936-12564588 | |
| BM658362, BG835786 |  |  |  |  | 27 | 12583569-12582694 | |
| BF046901 | 08H21 | 27_2 | G | G | 27 | 8746067-8746585 | |
| U57651 | 12L18 | 27_3 | G | R | 27 | 785268-741659 | |
| CA798822 | 26P13 | 28_1 | − | O | 28 | 10206607-10207197 | |
| BF707439 | 17D24 | 28_2 | − | G | 28 | 8035768-8039311 | |
| BE015381 | 10I15 | 28_3 | R | C5 | 28 | 6686161-6684499 | |
